# Structural motifs of gold cluster anions with 17 to 69 atoms

**DOI:** 10.1038/s41467-026-71649-9

**Published:** 2026-04-10

**Authors:** Andrés Aguado, Pablo Álvarez-Zapatero, Oleg Kostko, Bernd von Issendorff

**Affiliations:** 1 Departamento de Física Teórica, Atómica y Óptica, University of Valladolid, Valladolid, Spain; 2 Physikalisches Institut, Universität Freiburg, Freiburg, Germany; 3 FMF, Universität Freiburg, Freiburg, Germany

**Keywords:** Chemical physics, Structure of solids and liquids, Nanoparticles

## Abstract

Gold has very special bonding properties, which is mainly due to strong relativistic effects influencing its electronic system. Gold clusters therefore, exhibit a large variety of structures not seen for other metallic systems. However, a reliable identification of these structures, which usually is based on a comparison of simulated and measured spectra, has been difficult as calculations often could not reproduce the measurements even if the correct structural motif was assumed. Here we show that optimized DFT calculations can yield a close to perfect agreement between measured and simulated photoelectron spectra, which in combination with extensive structural searches permitted us to identify geometries of cluster anions with 17 to 69 atoms. Many of these turn out to be highly symmetric, demonstrating that the special bonding properties of gold do not favor amorphous structures. These results provide a basis for a more precise treatment of gold nanostructures.

## Abstract (web summary)

A combination of DFT calculations, comprehensive structural searches and high-resolution photoelectron spectra unveils a broad variety of often highly symmetric structural motifs of gold cluster anions with a few tens of atoms.

## Introduction

The metal gold is a fascinating material. One obvious reason is its resistance to corrosion, due to which it has been used for precious jewelry for thousands of years. For the same reason, it is nowadays used for, e.g., sensitive electrical contacts or—in the form of nanoparticles—as markers in biology^1,2^. It is also very special in its mechanical properties, as it can be hammered to sheets just 100 nm thick or can form wires of single atom width in break junctions^3^. The remarkable activity of supported gold clusters^4,5^ as low-temperature catalysts demonstrates that this metal also exhibits unexpected properties on the nanoscale. Many of these extraordinary properties are due to relativistic effects^6^, which induce a significant contraction of *sp* − orbitals, enhance *sd* − hybridization, and produce aurophilicity^6^ and abnormally high electronegativity values compared to other metals. In fact, gold is the “most relativistic” element below Fermium^7^, making it an interesting object of study to understand the influence of relativistic effects on material properties.

As clusters offer a very specific approach to study materials, it is no surprise that gold clusters have attracted a lot of interest—they are one of the most intensely studied cluster systems. Experimental studies on them encompass, e.g., mass spectrometry^8^, photofragmentation^9,10^, electron diffraction^11^, ion mobility^12^, infrared spectroscopy^13^, as well as photoelectron spectroscopy^14–19^, which is one of the most powerful techniques to study size-selected clusters in the gas phase. The photoelectron spectrum (PES) yields direct information about the electronic structure of the cluster, which is a very sensitive structural fingerprint. In combination with theory, it therefore also allows determining the geometric structure of the cluster. By such experimental and theoretical studies, it has been found that gold clusters can adopt a wealth of structural motives, reaching from planar structures up to surprisingly large sizes (*N* = 12 for anions)^20–23^, over cages around size 16 and pyramidal fcc lattice fragments at size 20 to hollow structures up to size 26. For larger sizes, the results often hinted at more amorphous structures^19^.

Nevertheless, despite this tremendous amount of work, there are still many open questions, which up to now were mostly due to the difficulty to perform precise and efficient first-principles calculations on gold clusters. Although fairly high-resolution photoelectron spectra have been available for many years, the theoretical electron density of states (EDOS) obtained in density functional theory (DFT) calculations could not match the spectra with sufficient accuracy. Accurate thermochemistry has also been a fundamental issue. Most previous attempts^18,19,24–29^ did not reach full consistency between theory and experiment: for many cluster sizes, a good EDOS/PES match could only be obtained using the EDOS of a highly excited isomer instead of that of the theoretical global minimum (GM) structure, thus casting doubts on the correctness of the structural assignment. In order to obtain a sound and definitive assignment of structural motifs, it is indispensable that theory provides both accurate electronic properties and thermochemistry. Many of the conclusions that have been established in recent years, of which some seemed to corroborate the old claim that medium-sized gold clusters tend to be amorphous^30–34^, might therefore not be correct and need to be revisited with more accurate calculations.

In this work, we identify a DFT level of theory that accurately describes both the electronic structure and the thermochemistry of gold cluster anions. We thereby succeed in reproducing the entire structure of well-resolved photoemission spectra to an unprecedented accuracy, in most cases exclusively employing the EDOS of the most stable structure (or of the two most stable ones) identified by theory, thus reaching full consistency between experiment and theory. Dispersion interactions, explicitly non-local exchange interactions, and in some cases also spin-orbit interactions, are found to be essential to obtain accurate thermochemistry, while the latter two are always needed to obtain the correct EDOS. The quality of the EDOS/PES match obtained is uniformly good in a wide size range of ${Au}_{N}^{-}$ cluster anions (*N* = 17−69), thus providing a convincing and definitive assessment of the structural evolution of small and medium-sized gold clusters. Our results reveal several new and well-defined structural patterns in gold clusters. Most of the GM structures are based on clearly identifiable structural motifs, and many have a high point-group symmetry. These results can be expected to have a profound influence on future research on gold clusters and nanoparticles.

## Results

### Identification of the optimum functional

We have performed extensive benchmark DFT calculations on a variety of gold systems, ranging from isolated atoms over neutral and charged clusters up to the bulk, in order to test a broad range of functionals (see the “Methods” section). It turned out that the simple and computationally rather inexpensive PBEsol functional already yields very accurate bond lengths and reasonable relative energies of structural isomers, which makes it the functional of choice for structure screening runs. The most accurate functional identified is the PBE0 hybrid with explicit spin-orbit and dispersion corrections; it not only provides highly reliable relative energies, but also reproduces the electronic density of states accurately.

**Fig. 1:**
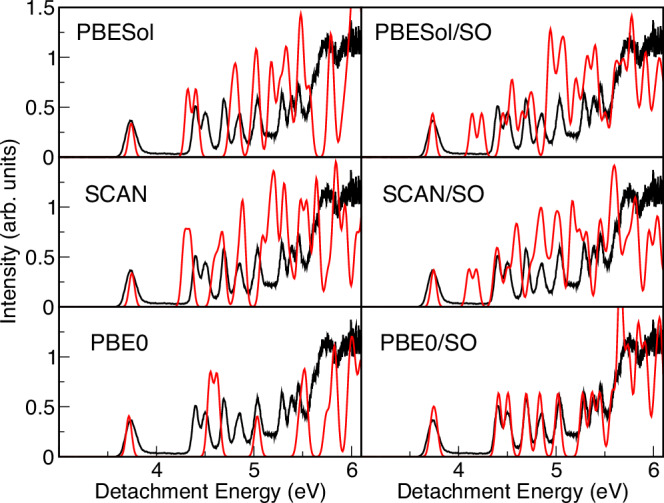
Benchmarking the accuracy of DFT functionals. Comparison of the EDOS (red lines) calculated with different functionals and the experimental photoelectron spectra data (black lines) for ${Au}_{19}^{-}$ (cluster geometry relaxed at the PBEsol level). The different rows display, from top to bottom, representative examples of the performance of GGA, meta-GGA, and hyper-GGA rungs of the DFT Jacob’s ladder^69^. The two columns show the effect of spin-orbit (SO) interactions on the obtained EDOS.

As a test case for the spectroscopic assessment, we used the cluster ${Au}_{19}^{-}$, as it has a highly structured photoemission spectrum and a well-known GM structure: a pyramid with a missing vertex^19^. Figure 1 compares the theoretical EDOS calculated by representative functionals to the experimental PES. We find that none of the GGA or meta-GGA functionals can produce a good match to the experimental results, irrespective of the inclusion of spin-orbit effects. PBEsol^35^ is chosen as the reference GGA functional because it produces very accurate geometries. SCAN is considered a sort of definitive meta-GGA functional^36^, yet the SCAN EDOS does not improve much over GGA results. Only a hyper-GGA functional such as PBE0 with explicit spin-orbit effects can reproduce the PES with high accuracy (as already has been observed by Wang and coworkers^19^). Other hybrids also produce a good match (see the Supplementary Information), but the original PBE0 hybrid provides the highest-quality one. The EDOS/PES match obtained is of unprecedented accuracy, with nine peaks in the PES perfectly reproduced by the calculations, and with even the onset of the “d-band”, the high density of states formed mainly from the atomic 5d orbitals, correctly positioned at about 5.6 eV. We ascribe the quality of this match to both the functional used and the highly accurate bond lengths as determined at the PBEsol level.

This test case demonstrates that if the correct cluster geometry has been found, one can expect to have a close to perfect match between the measured photoelectron spectrum and the calculated EDOS, which represents a stringent test of the putative geometric assignment. This motivated us to perform an intensive search for the ground state structures of gold cluster anions in the size range from 17 to 70 atoms; representative examples of the different structural families identified are presented in this report. We employed a multi-step computational protocol for the search, as detailed in the Methods section.

In the following, we will discuss the different cluster structures found.

### Comparison of simulated and measured photoelectron spectra

**Fig. 2:**
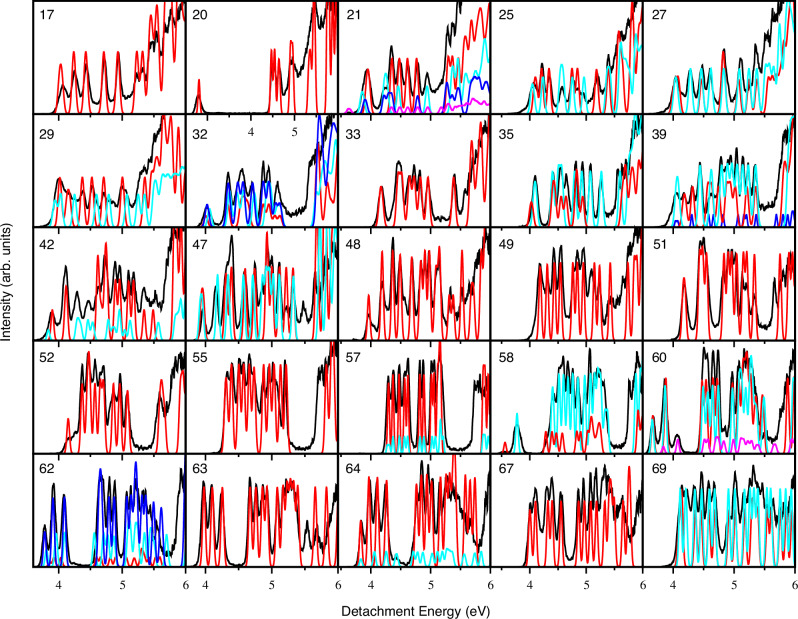
Comparison of the theoretical EDOS and experimental photoelectron spectra for representative sizes. Black curves: experimental signal; red curves: the EDOS of the putative GM structure. Only in cases of near-degeneracy, the cyan and blue curves display the EDOS of the first and the second excited isomer. The magenta curve for ${Au}_{21}^{-}$ is the EDOS of the fifth excited isomer, the only one matching a tiny low-energy peak in the experimental spectrum. Also for ${Au}_{60}^{-}$ the magenta curve indicates the EDOS of the fifth isomer, the lowest one matching the third peak. The GM structures used in the calculations and some competitive isomers are shown in Fig. 3; additional structures are presented in the Supplementary Information. The EDOS have been rigidly shifted to align the theoretical VDE with the experimental VDE values. When several isomers contribute to a given spectrum, their VDE values are shifted by exactly the same amount. See the Supplementary Information for the theoretical VDE values.

Figure 2 shows examples of the EDOS/PES match for the lowest-energy structures identified.

The experimental spectra are plotted with black lines, the red lines indicate the EDOS of the lowest-energy isomer, while the cyan and blue lines show those of the second and third lowest ones. In most cases, the calculated EDOS of the putative ground state structure (or a combination of those of the lowest and the next higher isomer) is in excellent agreement with the measured photoelectron spectrum—one has to emphasize that such a degree of agreement is unprecedented in cluster physics. For some sizes, however, the EDOS of the second lowest isomer (or a combination of those of the second and third isomers) gives a better match to the experiment. In all of these cases, the second lowest isomer is energetically close to the ground state and of lower symmetry. This leads to an entropic advantage of the second isomer (or the second and the third, as in the case of cluster size *N* = 39) over the ground state. Although lower in energy, the ground state structures are less abundant than the higher lying ones; see the Supplementary Information for a more detailed discussion. Also presented in the Supplementary Information are comparisons of the measured spectra with the EDOS of higher lying isomers, as well as a quantitative assessment of the EDOS/PES match. One should mention that for larger clusters (with a larger number of internal atoms), the reproduction of the onset of the d-band is, in general, less good than for size *N* = 19; it is a common problem of DFT that delocalized and more localized electron states (here: the s/p-band and the d-band) usually cannot be described with the same quality^37^. In some cases, the binding energy of peaks close to the d-band onset is overestimated; these peaks correspond to states with a rather large degree of d-orbital admixture.

In any case, the quality of the EDOS/PES match yields strong evidence that the correct GM structures were located for all sizes shown.

### Identification of structural motifs

**Fig. 3:**
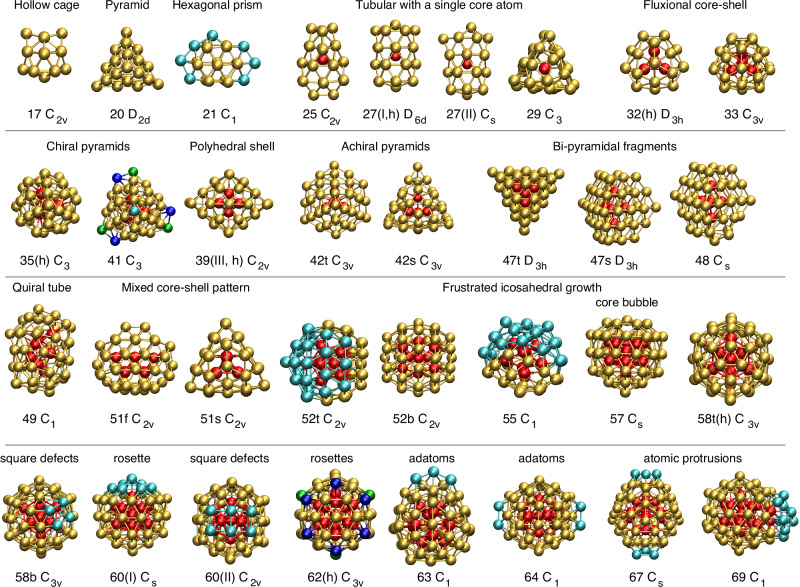
Evolution of structural trends and identification of the main structural motifs for gold cluster anions in the size range *N* = 17−69. The cluster size and the approximate point group symmetry of the structure are given below each image; the letters f,s,t,b indicate front, side, top, and bottom views in cases where more than one view is shown. The GM structures that are ''hidden” in the experiment due to an entropic advantage of isomers with lower symmetry are marked with the letter h (for size *N* = 39 isomer III is practically degenerate with isomers I and II, but ''hidden” due to its higher symmetry). The structure of size *N* = 41 is not the GM, but represents a structural motif seen for smaller sizes, e.g., *N* = 35. Red color is used for core atoms, while blue and green colors highlight some structural features at the cluster surface, which are discussed in the text. Additional isomers are presented in the Supplementary Information, as well as the calculated relative energies of the isomers. Coordinates of all structures are provided in this paper in Supplementary Data 1.

Based on these results, we now describe the main trends in the structural evolution of gold cluster anions, focusing on representative cluster sizes. A detailed description of all structures in the studied size range will be offered in separate full-length papers. Some of the structures presented here have been identified before, namely those of the clusters with 17^24^, 20^18^, 21^26^, and 33^38^ atoms. Other structures (*N* = 32, 35, 47, 49, 55−60) are related but not identical to published ones^38–40^; here we often identify more symmetric (and energetically favorable) isomers. For the remaining thirteen sizes, we present new GM geometries. All GM structures (as well as some excited isomers) are shown in Fig. 3; the structures of all other isomers contributing to the PES are presented in Supplementary Fig. 5.

As stated above, for size *N* = 17 we confirm previous findings^19^: the GM structure is a hollow pentagonal prism, with its five equatorial facets covered with adatoms. The adatoms are not all equivalent, as three of them are located at a shorter distance from the center, producing local distortions of the cage and creating additional bonds with neighboring adatoms. The GM structures for *N* = 19−20 adopt the well-known compact tetrahedral shape. ${Au}_{20}^{-}$ undergoes a slight distortion away from the perfect tetrahedral symmetry due to its unpaired valence electron; its exact symmetry is D_2*d*_.

For clusters with *N* = 21 atoms, the GM structure is a quite planar bi-layer structure without internal atoms. It is composed of a hexagonal prism, with adatoms decorating its equatorial facets (similar structures can be found up to size *N* = 25). At least three additional isomers contribute to the spectrum; of these, isomers II and III (shown in the Supplementary Information) are quite similar to the GM. Isomer II can be produced from the GM by just moving one atom to a different equatorial position; isomer III can be produced from isomer II by twisting the hexagonal prism into a hexagonal antiprism. The small peak at lower binding energy can best be explained by a small contribution from isomer V, the Au_20_ tetrahedron with one atom added.

All GM structures for the sizes *N* = 25, 27 and 29 can be classified as atom-filled elongated tubular structures. Dispersion is crucial for obtaining the transition between structures without and with one core atom at size *N* = 25, because the hexagonal prismatic family is still competitive for *N* = 25, and contributes quite a bit to the spectrum (cyan curve). The tubes can be highly symmetric. As an example, the shell of ${Au}_{27}^{-}$ is a nanotube composed of four 6-atom rings in perfect antiprismatic stacking plus two cap atoms, producing a high D_6*d*_ symmetry. This tube has a low-energy excitation, also shown in Fig. 3, which lowers the symmetry to C_*s*_. The two structures are nearly degenerate, so that the C_*s*_ isomer is favored by configurational entropy and therefore more abundant than the GM already at slightly raised temperatures - one could therefore call the high symmetry structure a “hidden” ground state (indicated by the letter “h” in Fig. 3). Indeed, the PES could be fully explained only by the excited isomer (cyan line). As another example, ${Au}_{29}^{-}$ features a chiral tubular shell with a clear 3-fold rotational axis. Its second isomer, which contributes weakly, is a distorted version of the hexagonal prism, covered with 15 atoms. Hollow tubes, suggested as competitive in previous works^29^, are found here only at very high energies because they are strongly destabilized by the dispersion interactions. Compact layered fcc-like structures, such as bi-pyramids, are competitive but never become the GM structure.

Clusters with 32 atoms have a three-atom core in the shape of an equilateral triangle, which endows these clusters with a globally oblate shape. As strange as it may be for typical metal clusters, ${Au}_{N}^{-}$ anions directly transition from structures with a single core atom to structures with 3 core atoms, i.e., structures with a dimer core are not stable. The GM structure of ${Au}_{32}^{-}$ has a high symmetry; Fig. 3 shows that its shell preserves the full D_3*h*_ symmetry of the cluster core. In the shell, an open square facet forms on top of each core atom, a characteristic trend that will be observed in many of the larger gold clusters. Another characteristic of such core-shell structures is their fluxionality: the core can adopt several stable orientations through a nearly free rotation with respect to the shell, which produces several nearly degenerate local minima. The most stable version is always the most symmetric one, but lower-symmetry versions have a higher configurational entropy and may easily become more abundant in an experimental sample, so that several isomers will contribute to the photoemission spectrum. For ${Au}_{32}^{-}$ at least two of the lower symmetry versions contribute significantly to the spectrum, making the D_3*h*_ structure again a “hidden” ground state.

${Au}_{33}^{-}$ also has a highly symmetric core-shell structure, which is, however, less fluxional because of its larger four-atom tetrahedral core. Size 35 adopts the form of a twisted trigonal pyramid with a four-atom tetrahedral core. A complete twisted pyramid can be built with 41 atoms and is shown in Fig. 3, although it is not the GM for ${Au}_{41}^{-}$. It has one tip atom, which is just 3-fold coordinated and highlighted in light blue, and 6 basal corner atoms displayed in green and dark blue colors: the green ones are coordinated to four neighbors and, once removed, leave the dark blue ones as 4-fold coordinated as well. GM structures or nearly degenerate isomers in this size range are obtained by simply removing some of these low-coordinated atoms from the complete pyramid, apart from local relaxations around the vacancies thus created. As an example, ${\rm{Au}}_{35}^{-}$ results from removing the six basal atoms and thus retains the full C_3_ symmetry. In fact, many GM structures in this size range are fragments of the twisted 41 atom pyramid, i.e., they all display a well-defined and systematic growth pattern.

For ${\rm{Au}}_{39}^{-}$ a new structure becomes competitive to the twisted pyramids. It has an even higher symmetry; its shell can be seen as formed from flat triangular Au_10_ and Au_6_ units. At the PBE0-D3 level, it lies only 7 meV above the GM and should therefore contribute significantly to the experimental spectrum; nevertheless, this high-symmetry structure is another example of a hidden isomer; the PES for ${\rm{Au}}_{39}^{-}$ can be fully explained by the second and the third isomers, both twisted pyramids.

For ${\rm{Au}}_{42}^{-}$ an achiral version of the triangular pyramid forms the GM. Achiral pyramids have a slightly less compact packing in their shell, so more atoms can be allocated to it, avoiding the generation of low-coordinated adatoms. A complete pyramid is the GM structure of ${\rm{Au}}_{42}^{-}$, shown in Fig. 3 in top and side views. For ${\rm{Au}}_{42}^{-}$ additionally, an icosahedral fragment with 4 core atoms (obtained by removing atoms from one side of the full 55-atom Mackay icosahedron, see Supplementary Fig. 5) is competitive with the achiral pyramid, both structures together matching the photoemission spectrum.

The GM structures of the sizes 47 and 48 are bi-pyramidal fragments obtained by removing atoms from the complete 55-atom trigonal bi-pyramid, which in turn results from merging two Au_35_ tetrahedra. The core is a 5-atom trigonal bi-pyramid in both cases. The atomic structure of these sizes is perfectly ordered, so high symmetries can emerge, the highest being the D_3*h*_ GM of ${\rm{Au}}_{47}^{-}$. The only distortions observed include: a slight rounding of the ideally planar facets, whereby the most coordinated surface atoms undergo an outward relaxation, and a significant local relaxation around the adatom in the GM of ${\rm{Au}}_{48}^{-}$, which increases its coordination number. Isomer coexistence is generally not an issue in this size range, but there is a very interesting near-degeneracy of two spin states for ${\rm{Au}}_{47}^{-}$: the most symmetric D_3*h*_ structure favors a spin triplet state; in the corresponding singlet state, the cluster undergoes a slight Jahn-Teller distortion down to C_2*v*_ symmetry, which has a significant effect on the EDOS. Both spin isomers are needed to reproduce the PES.

Even if the bi-pyramids remain somewhat competitive for the next sizes, the dispersion interaction induces a sudden increase in core size; the most stable clusters with *N* = 49, 51 feature 7-atom and 8-atom cores. Surprisingly, 6-atom cores are never stable in gold cluster anions. ${\rm{Au}}_{49}^{-}$ can be seen as an elongated chiral tube, where the shell atoms grow around the core by forming a spiral or helical chain. ${\rm{Au}}_{51}^{-}$ is a special cluster that stands out in the structural evolution. It features a layered fcc-like core surrounded by a rounded polyhedral shell, so core and shell seem to grow quite independently of each other. The cluster as a whole has a respectable C_2*v*_ symmetry, but the side view in Fig. 3 shows that the shell alone would even have a higher C_3*v*_ symmetry.

At size *N* = 52, a new and well-defined growth pattern is established and maintained at least up to size *N* = 70, so no gold cluster in this size range should be considered truly amorphous. The new packing rules emerge from a competition between two incompatible but strong features: metallic bonding tends to favor Mackay icosahedral packing in this size range, but the stiffness of the gold bonds strongly penalizes the bond strain that occurs in complete icosahedral shells^41^. Gold clusters, therefore, adopt what we term a frustrated icosahedral growth, where the structures are dominantly close to perfectly icosahedral, but release the stress through local atomic rearrangements in a small region of the cluster. The rich structural diversity observed in the size range *N* = 52−70 is the result of the many different solutions that nature finds to avoid the bond strain.

For size *N* = 52 the GM structure is based on a 55-atom Mackay icosahedron, which is flattened on one side, but nearly perfectly icosahedral on the other side. In Fig. 3, we show both side and bottom views of this cluster. In order to better visualize the icosahedral packing in its top side, we have highlighted the 16-atom pentagonal caps that occur around each vertex of a 55-atom Mackay icosahedron. Incomplete icosahedral fragments are not necessarily strained, and we indeed observe that the radial distance between each pentagonal vertex atom and the atom immediately below is essentially the same as its distances to the five neighboring atoms in the surface. The stress is released by the approximately flat terrace, a solution that provides similar bond lengths everywhere in the cluster. The core of this and larger clusters is itself an icosahedral fragment, which we term Ih_9_ for *N* = 52 and Ih_10_ for *N* = 55. Here, Ih_9_ stands for a 13-atom icosahedron from which four contiguous atoms have been removed. The Ih_9_ core has C_2*v*_ symmetry; for ${\rm{Au}}_{52}^{-}$ the congruent shape of the shell respects that symmetry. For ${\rm{Au}}_{55}^{-}$ the Ih_10_ core has C_3*v*_ symmetry, but due to the flattening of one side of the icosahedron the overall symmetry of the cluster is only C_1_. It is interesting to note that size *N* = 55 in some previous works was considered a sort of paradigmatic example for the tendency of gold clusters towards amorphization^30,42^, yet it clearly belongs to the family of flattened icosahedra despite its low symmetry.

With ${\rm{Au}}_{57}^{-}$ we enter a size interval featuring highly ordered, symmetric, and more spherical structures, as we approach the formation of a full icosahedral core. ${\rm{Au}}_{57}^{-}$ has an Ih_12_ core: an icosahedron with a missing vertex, a feature we term a “core bubble”, and which can be seen in the top part of the cluster in Fig. 3. 11-atom cores are not stable for gold cluster anions. The very spherical shape of the GM structure is favored by the electronic shell closing occurring for 58 electrons. The structure is close to perfectly icosahedral and without strain in its bottom part, but reconstructs its upper side in order to release the stress otherwise occuring. Here, we identify two new mechanisms for stress release: first, the incorporation of further atoms into the shell. This is what one sees in the upper part of the shell, where the 10-atom and 5-atom rings, that would occur in a Mackay shell, are substituted by 11-atom and 6-atom rings; second, the formation of a bubble, which also helps to achieve optimal bond distances everywhere. In exactly the same way as the shell adapts to close-by core atoms by opening square or even pentagonal defects, it adapts to the bubble with defects that locally increase the surface packing density, in this case with the 7-atom facet located right above the bubble. We have explicitly checked that the bubble is stable only at this subsurface site, i.e., bubbles at other subsurface sites or more deeply buried bubbles produce highly excited isomers. The structure is evidently ordered even if the presence of the 11-atom and 6-atom rings destroys the 5-fold symmetry, due to which the global symmetry is only C_*s*_. There is a second isomer of a very similar structure (see Supplementary Fig. 5), which is almost degenerate with the GM and therefore should contribute to the spectrum; its EDOS, however, is so similar to that of isomer I that it cannot be distinguished.

For *N* = 58, structures with Ih_12_ and Ih_13_ cores coexist, while clusters with *N* = 60−63 atoms clearly have a complete Ih_13_ icosahedron in their core. The GM structure of ${\rm{Au}}_{58}^{-}$ has C_3*v*_ symmetry and thus preserves many of the symmetries of its Ih_13_ core. On its top side (first view in Fig. 3) there are six complete 16-atom icosahedral caps; its bottom side displays nine square defects symmetrically arranged around the 3-fold axis (one of these square defects is highlighted in Fig. 3). Its second isomer has a Ih_12_ core and only C_1_ symmetry; it is similar to the isomers I and II of ${\rm{Au}}_{57}^{-}$, with one additional atom added to the shell. This isomer, in fact, dominates the spectrum, as the high symmetry GM is suppressed by entropy again. ${\rm{Au}}_{60}^{-}$ has two nearly degenerate GM structures. One of them (isomer II), with C_2*v*_ symmetry, presents again several square defects in its shell; the other one has a perfectly triangulated surface, which resembles an icosahedral shell with several atoms inserted. While rings with 1-5-10-10-10-5-1 atoms are stacked in the outer shell of a perfect 55-atom Mackay icosahedron, the shell of ${\rm{Au}}_{60}^{-}$ presents a 1-6-11-11-11-6-1 stacking sequence. A capped six atom ring, also known as a rosette defect^43^, is highlighted at the top. A third isomer contributes to the spectrum, which is a slightly distorted version of the GM. ${\rm{Au}}_{62}^{-}$ has a C_3*v*_ shell without any square defects and with exactly 12 pentagonal vertices, six of which are of perfect Mackay type. On the other side of its shell (shown in Fig. 3), seven additional atoms are incorporated (as compared to a perfect Mackay icosahedron), which generate six neighboring pentagonal vertices (indicated by dark blue atoms) and three rosettes (green atoms), all symmetrically allocated around the 3-fold axis. This highly symmetric structure is, however, another example of a hidden GM; the two next higher isomers dominate the spectrum. Isomer II is similar to isomers I and III of ${\rm{Au}}_{60}^{-}$, with two more atoms added into the shell; isomer III is the very symmetric isomer II of ${\rm{Au}}_{60}^{-}$, with two atoms added on top of the shell on one side. For larger clusters, it finally becomes impossible to allocate all the additional atoms in a single shell; consequently, some clear adatoms appear. The GM of ${\rm{Au}}_{63}^{-}$ has an Ih_13_ core, a mostly icosahedral shell consisting of 47 atoms, and three adatoms attached to the shell. The GM of ${\rm{Au}}_{64}^{-}$ is again obtained from the C_2*v*_ isomer of ${\rm{Au}}_{60}^{-}$ by capping square facets with adatoms on two opposite sides. There is an associated small distortion, so its precise symmetry is C_1_, but it is very close to the C_2*v*_ symmetry of the parent cluster. Another isomer probably contributes, which is the GM of ${\rm{Au}}_{60}^{-}$ capped with a string of 4 atoms.

The bigger clusters tend to depart from a spherical shape, but continue to belong to the frustrated icosahedral family. Size *N* = 67 has an Ih_14_ core (an icosahedron plus one adatom), producing an elongated global shape, while size *N* = 69 already features an Ih_15_ core. The adatoms tend to agglomerate and form localized atomic protrusions on otherwise Mackay icosahedral shells. The protrusion itself can be somewhat amorphous, as in the two lowest isomers of ${\rm{Au}}_{69}^{-}$; this lowers the global symmetry to C_1_, even if the biggest part of the shell is icosahedral. It can also be ordered, as for the GM of ${\rm{Au}}_{67}^{-}$ with its two small protrusions. We expect that this type of growth will remain stable over a wider size range than explicitly studied here, though probably new stress release mechanisms will become operative as well.

## Discussion

**Fig. 4:**
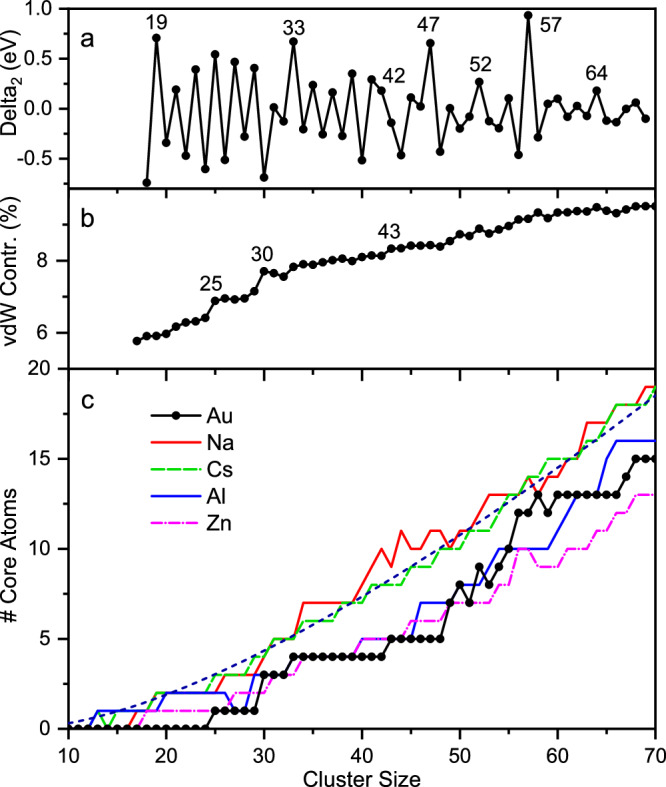
Size dependence of gold cluster anion properties. **a** Negative discrete second derivative of the total cluster energies (2*E*_*n*_ − *E*_*n*−1_ − *E*_*n*+1_), which is a measure for relative cluster stability. **b** Contribution of the dispersion interaction to the total binding energy (in percent). **c** Core size (number of fully enclosed atoms). Additionally to the data for gold cluster anions obtained in this work (black dots), core sizes of cluster anions are shown for the metals sodium (red line)^45^, cesium (green line, dashed)^46^, aluminum (blue line)^47^, and zinc (magenta line, dot-dashed)^49^. The dark blue dashed line indicates the core size for idealized spherical clusters (see the Supplementary Information for details).

The assignment of the geometric structures allows one now to examine some peculiarities in the size dependence of the gold cluster properties. For completeness, we include here results for all sizes between 17 and 70. Figure 4a shows the second derivative of the cluster binding energies with respect to size, which yields a measure of the cluster stabilities. The smaller sizes exhibit an odd-even oscillation typical for monovalent simple metal clusters; odd sizes (with an even number of electrons due to the negative charge) are usually electronically closed shell and therefore more stable than even ones. Especially stable are magic sizes with closed free electron shells, like sizes *N* = 19 (configuration 1*S*^2^1*P*^6^1*D*^10^2*S*^2^ in the spherical Jellium model^44^) and *N* = 33 (configuration 1*S*^2^1*P*^6^1*D*^10^2*S*^2^1*F*^14^). Beyond size 40, the oscillations are much less pronounced; here, even some even-sized clusters have a higher stability, like *N* = 42, 52, 64, which must be due to their especially stable atomic structure. Size 57 sticks out; it does not only have a closed electronic shell (fully filled 1G), but also a well-ordered atomic structure, and therefore can be seen as double magic. Size 47 is also very stable. In this case, the cluster has a very symmetric atomic structure but no electronic shell closing. These results underline the importance of the atomic arrangement already for medium sized gold clusters. Figure 4b presents the contribution of the van-der-Waals interaction to the total binding energy (in percent). One can see that in the size range considered here, it already amounts to values between 6% and 9.5%, which is significant. It slowly increases with size, and converges to a value of 21% of the cohesive energy in the bulk limit (see Table 1 in the “Methods” section). Especially for the smaller sizes, one can clearly recognize the effect of the increase of the core size, which increases at size *N* = 25 from 0 to 1, at size *N* = 30 from 1 to 3, and at *N* = 43 from 4 to 5. The increase of the core size increases the average coordination number and therefore the dispersion interaction; in fact, the dispersion interaction is crucial to determine the correct core size.

**Table 1:** DFT calculations employing the PBE0 hybrid functional without and with inclusion of dispersion corrections (D3) and explicit spin-orbit effects (SO), benchmarked against relativistic coupled cluster calculations and experimental values

System	Property	PBE0	PBE0-D3	PBE0-SO-D3	R-CCSD(T)	Exp.
Au (atom)	IP[eV]	9.25	9.25	9.24	8.98^b^/9.21^c^	9.23^a^
	EA[eV]	2.03	2.03	2.05	2.12^b^	2.31^a^
Au_2_	*R*_*e*_[Å]	2.514	2.507	2.499	2.51^b^/2.488^e^	2.472^a^
	*D*_*e*_[eV]	2.097	2.150	2.215	2.12^b^/2.19^e^	2.302^a^
	*ω*_*e*_[cm^−1^]	187	189	191	186.9^e^	191^a^
	*vIP*[eV]		9.37	9.35	—	9.50^f^
	*aIP*[eV]		9.18	9.17	9.15^b^	9.16^g^
	*aEA*[eV]		1.80	1.81/2.06^k^	1.83^b^	1.94^b^
${\rm{Au}}_{2}^{+}$	*R*_*e*_[Å]	2.628	2.623	2.620	2.639^b^	
	*D*_*e*_[eV]	2.178	2.219	2.270	1.95^b^/2.02^e^	2.21 ± 0.21^l^
${\rm{Au}}_{2}^{-}$	*R*_*e*_[Å]	2.631	2.626	2.616	2.632^b^	2.582^b^
	*D*_*e*_[eV]	1.879	1.920	1.955	1.83^b^	1.94^b^
	*vDE*[eV]		1.84	1.83/2.10^k^		2.01^m^
Au_3_	*D*_*e*_[eV]	3.159	3.330	3.630	3.25^b^	3.80^h^
	*vIP*[eV]		6.91	6.95	6.84^b^/6.88^d^	7.50^f^
	*aIP*[eV]		6.82	6.79	6.75^b^	7.27^g^
	*aEA*[eV]		3.38	3.31/3.59^k^	3.62^b^	
${\rm{Au}}_{3}^{+}$	*R*_*e*_[Å]	2.634	2.629	2.626	2.631^b^	
	*D*_*e*_[eV]	5.529	5.694	5.808	5.49^b^	
${\rm{Au}}_{3}^{-}$	*R*_*e*_[Å]	2.577	2.571	2.568	2.573^b^	
	*D*_*e*_[eV]	4.505	4.675	4.790	4.73^b^	
	*vDE*[eV]		3.53	3.57/3.84^k^	3.73^b^	3.87^m^
Au_4_	*D*_*e*_[eV/atom]	1.177	1.453	1.525	1.557^d^	
	*vIP*[eV]		7.73	7.77	7.78^d^	7.82 ± 0.18^i^
						8.60^f^
Au_5_	*D*_*e*_[eV]	1.205	1.615	1.678		
	*vIP*[eV]		7.29	7.33	7.36^d^	8.00^f^
Au_3−8_	MAE(*R*_*e*_)[Å]	0.020	0.014	0.009	0.00^d^	
${\rm{Au}}_{12}^{-}$	*E*(3*D*) − *E*(2*D*)[eV]	0.556	0.059	0.005		~0^n^
Au(solid)	*a*[Å]	4.096	4.049	4.039		4.059^j^
	*E*_*coh*_[eV]	2.83	3.64	3.80		3.83^j^

The benchmark covers the isolated gold atom, the neutral and ionic states of dimer and trimer, small sized clusters and the bulk crystalline state, and includes several geometric, electronic and energetic quantities: *vIP*: vertical ionization potential; *aIP*: adiabatic ionization potential; *aEA*: adiabatic electron affinity; *vDE*: vertical detachment energy for anions; *R*_*e*_: equilibrium distance; *D*_*e*_: dissociation energy; *ω*_*e*_: vibrational frequency; *a*: bulk lattice constant; *E*_*coh*_: bulk cohesive energy; *E*(3*D*) − *E*(2*D*): energy difference between the nearly-degenerate three-dimensional and planar isomers of ${Au}_{12}^{-}$; finally, MAE(*R*_*e*_) refers to the mean absolute deviation of interatomic distances from the R-CCSD(T) reference values^57^ for clusters with between 3 and 8 atoms.

^a^Data taken from ref. ^6^.

^b^Data taken from ref. ^59^. These R-CCSD(T) calculations do not include explicit spin-orbit effects.

^c^Data taken from ref. ^60^, obtained from a fully relativistic (four-component) open-shell equation-of-motion CCSD(T) method.

^d^Data taken from ref. ^57^. CCSD(T) geometry optimizations were performed at the scalar relativistic level with a small core (19 valence electrons per gold atom).

^e^Data taken from ref. ^61^.

^f^Experimental vIP obtained by electron impact ionization^62^.

^g^Experimental aIP determined by charge-transfer bracketing^63^.

^h^Data taken from ref. ^64^.

^i^Experimental vIP obtained by photoionization^65^.

^j^Data taken from ref. ^66^.

^k^The second vDE value is extracted from the difference of the calculated dissociation energies of the neutral and the anionic system, adding the experimental electron affinity of the atom instead of the calculated one. It demonstrates that the vDE errors are mostly systematic and originate in the error of the isolated atom electron affinity.

^l^Experimental *D*_0_ value obtained by collision induced dissociation^67^; converted to *D*_*e*_ assuming *ω* = 149 cm^−1^ ^67^.

^m^This work.

^n^Estimated from the coexistence of the two isomers^68^.

Figure 4c finally shows this size dependence of the core sizes. For comparison the core sizes for four other systems are shown, for sodium, cesium, aluminum and zinc clusters, as determined earlier^45–49^. Additionally shown is the core size expected within densely packed spherical particles (see the Supplementary Information for a derivation); in fact, this curve matches core sizes in closed shell icosahedral structures almost perfectly. One can see that sodium and cesium clusters follow this curve closely, due to their icosahedral growth scheme. Aluminum, zinc, and gold clusters exhibit much smaller core sizes, but for very different reasons. Aluminum clusters tend to form fcc-structures with large planar facets already for rather small sizes; the planar facets lead to a large number of surface atoms and consequently to smaller core sizes^47^. Zinc clusters, on the other hand, tend to adopt rather spherical shapes, but with a significant gap between the outermost layer of atoms and the core—this also leads to a smaller core size^48,49^. Gold clusters again behave differently: they tend to form atom-sized bubbles within the cluster structures, as well as planar facets on one side of otherwise spherical particles; both of these tendencies reduce the number of atoms in the core. This clearly demonstrates the difference between gold and simpler metals.

We have found that these peculiarities are due to a delicate balance between non-local exchange, dispersion, and spin-orbit effects. While attractive dispersion effects systematically stabilize structures with a denser atomic packing, non-local exchange effects do exactly the opposite, so accounting for both effects in a transferable and accurate way is essential to obtain, for example, the correct number of core atoms in the GM structures. The spin-orbit contribution to cluster stability is also essential for the determination of the GM structure for some sizes, but its effect is much less systematic or predictable (see the Supplementary Information for examples of the strong influence of these effects on the optimal geometry).

Our study is expected to have a significant impact on any future gold-related computational research. The more accurate level of theory assessed here should provide better results not only for gas phase clusters, but also for supported and ligand-protected versions of gold clusters, which are more relevant for practical applications.

## Methods

### Experimental methods

Gold cluster anions have been produced in a liquid nitrogen cooled magnetron sputter gas aggregation source, guided by and briefly stored in a room temperature radio frequency ion guide, before being injected into a double reflectron time-of-flight mass spectrometer. Here, a multi-wire mass gate between the two reflectors was used to select a given cluster size. The clusters were then rebunched by the second reflector, decelerated, and inserted into the interaction region of a magnetic bottle type photoelectron spectrometer, where they were irradiated by laser pulses from an ArF excimer laser, at a photon energy of 6.4 eV. Spectra were averaged over typically 30,000 shots, and constitute averages of the kinetic energy distributions of photoelectrons detached from roughly 10,000–100,000 clusters. The spectrometer was calibrated using the known photoelectron spectrum of Pt anions, resulting in an uncertainty of measured electron binding energies of less than 30 meV. The cluster temperature in this set of measurements is estimated to be about 200 K.

### Computational methods

#### Global optimization. Empirical potential and neural network

Our search for GM structures involves a hierarchical procedure with several independent stages. In the first one, extensive Basin Hopping (BH) global optimization runs^50^ are conducted to explore the potential energy landscape generated by a Gupta empirical potential (EP)^51^, which contains just two independent parameters when expressed in reduced units^48,49^:

1 $$E=\frac{1}{2}\mathop{\sum }\limits_{i}^{N}\left\{\mathop{\sum }\limits_{j\ne i}^{N}\chi \exp [{r}_{ij}]-{\left[\mathop{\sum }\limits_{i\ne j}^{N}\exp [-2\lambda {r}_{ij}]\right]}^{1/2}\right\}.$$

Here, *N* is the number of atoms in the cluster, and the *r*_*ij*_ denote interatomic distances. For physically meaningful potentials, *λ* ∈ (0, 0.5) and *χ* > 0. *λ* fixes the rate of spatial decay of the attractive many-body interaction relative to that of the pairwise repulsion, and controls the packing density of atoms or average coordination number: low *λ* values produce geometries with crowded cores; increasing the value of *λ* produces a steady increase in the ratio of surface to core atoms. *χ* is the ratio of the repulsive to the attractive energies at *r*_*ij*_ = 0, and essentially controls the stiffness of the bonds: lower *χ* values produce clusters with larger deviations of interatomic distances from the mean value; higher *χ* values lead to more uniform bond lengths. For sufficiently large *χ*, the potential always favors non-strained structures such as fcc fragments irrespective of the *λ* value. We refer the reader to the Supplementary Information for a description of more technical details, such as the choice of optimal parameters for gold, the number and length of the BH runs, the size of the stored data pool, or the protocol for selecting candidate structures from that pool for DFT re-optimization.

The BH runs based on the Gupta potential were able to identify the correct structural families for most ${\rm{Au}}_{N}^{-}$ clusters with *N* = 17−70 atoms. Nevertheless, for some cluster sizes, the Gupta potential failed in identifying structures that can explain the photoemission spectrum. Only for those sizes, we conducted additional BH runs using our recently developed neural network (NN) strategy^52^. Following the Behler-Parrinello approach^53^, we have trained a feed-forward NN potential to DFT energies and forces. This NN contains three hidden layers with a total of 4000 adjustable parameters, and is trained on a dataset comprising 9000 cluster configurations, ranging from 15 to 80 atoms, and evaluated at the PBE0-D3 level of theory. In its input layer, each cluster structure is represented not only by the original set of purely local functions (describing the radial and angular distribution of neighbors around every atom in the cluster^53^), but we also improved the structural description by adding a set of global features. These are the normalized principal inertia moments of the cluster, carrying information about the global cluster shape (spherical, prolate, oblate, etc.), which, according to jellium models, should be relevant to the stability of metal clusters. The use of global descriptors notably improves the accuracy of the NN potential, which reproduces PBE0-D3 energies within 3–4 meV/atom and PBE0-D3 atomic forces within 0.1 eV/Å. The NN potential was able to locate the GM for many sizes, where the Gupta potential failed to do so.

#### DFT calculations. Local optimizations

Around 400 isomers are locally re-optimized at the PBEsol level of theory^35^ for each cluster size. This functional has the virtue of providing very accurate cluster geometries at a relatively low cost (as discussed in the Supplementary Information), allowing massive optimization of a huge number of isomers even for big cluster sizes. Next, we perform single-point PBE0-D3^54,55^ calculations on the optimal PBEsol geometries for all isomers within an energy window of 0.2 eV (0.4 eV for sizes *N* < 26) above the PBEsol global minimum. The D3 dispersion correction has been implemented with Becke-Johnson damping parameters^56^. The single-point approximation is explicitly assessed by testing for a few sizes that optimal PBEsol and PBE0-D3 geometries hardly differ from each other (see the Supplementary Information). Finally, in the last step, we include spin-orbit effects by performing single-point PBE0-SO-D3 calculations on the 5–10 most stable PBE0-D3 isomers. Full technical details of these calculations are provided in the Supplementary Information.

#### Calculation of vertical detachment energies and simulation of the PES

Vertical detachment energies (VDE) of ${\rm{Au}}_{N}^{-}$ anions are calculated through a Δ − SCF calculation, that is, as the total energy difference between the anion and the neutral cluster, both clusters adopting the geometry of the anion. In order to simulate the experimental PES, we have broadened each line of the KS eigenvalue spectrum by using a Gaussian function of width 0.03–0.04 eV, which leads to a good agreement with experimental line widths. We have also rigidly shifted the EDOS on the energy axis in order to superpose and visually compare experimental and computational data in the same graph (Fig. 2). We emphasize that this is done only to help visualization of theoretical and experimental results within a single plot, and that only a global rigid shift was used to produce the EDOS/PES figures—the energy separation between peaks in the EDOS was not changed. Also, when comparing the EDOS of several isomers for a given size, the *same* global shift was applied to the VDE of all isomers, i.e., differences in the VDE values of the isomers are also reliably read off Fig. 2. The theoretical VDE values, as well as the magnitude of the energy shifts applied to each size, are given in Supplementary Table 4.

The assignment of correct structures is based on the energetic ordering provided by the PBE0-SO-D3 results and on an explicit comparison of the theoretical EDOS with the PES data. Notice that the two criteria do not need to be consistent with each other, i.e., it is not clear a priori that the most stable isomer according to theory is also the one with an EDOS that best matches the spectrum. As a result, only the second criterion has been used in most previous works^19^, as DFT energetics have been considered less reliable than DFT predictions of EDOS. The most important conclusion of this report is that PBE0-SO-D3 results fulfill both criteria.

#### Benchmark assessment

We have performed an extensive set of benchmark calculations ranging from the isolated gold atom, through neutral and charged states of small clusters with *N* = 2−12 atoms, to the bulk crystalline phase, using the VASP code. The results, summarized in Table 1, demonstrate that PBE0-SO-D3 calculations reproduce structural, vibrational, and energetic properties, in very good agreement with both experiment and relativistic coupled cluster calculations (see the Supplementary Information for additional results).

The equilibrium distance *R*_*e*_ of the dimer is within 1% of the experiment and R-CCSD(T) calculations. Dispersion and S-O effects are small for *R*_*e*_, although not negligible, and become larger for the dissociation energy. The S-O effect on stability becomes particularly important for the anion trimer, for example. The mean absolute error (MAE) of the atomic coordinates as compared to R-CCSD(T) results^57^ is below 1% for all clusters in the size range *N* = 3−8, and the energy differences between isomers compare equally well with R-CCSD(T) results (full details provided in the Supplementary Information). Clearly, both the structure and energetic properties of gold clusters are very accurately captured by the PBE0-SO-D3 level of theory. The dimer vibrational frequency is equally accurate.

Even if the dispersion contribution is small in the dimer, its relative magnitude increases with size, and accounting for it becomes completely crucial to get a uniformly good description of cohesion from the isolated atom to the bulk limit. As an example, the dispersion contribution to the binding energy evolves from 57 meV/atom for Au_3_ and 111 meV/atom for Au_8_, to 810 meV/atom in the bulk limit. It is the most decisive contribution to reproducing the near-degeneracy of 2D and 3D isomers of ${\rm{Au}}_{12}^{-}$ and the experimental cohesive energy of the crystalline solid.

Our calculations provide an ionization potential for the gold atom that is in perfect agreement with the experimental value; the vIP and aIP values of small gold clusters are in very good agreement with CCSD(T) results, which we take as the accuracy standard here (the scarce available experimental results differ a lot from each other, see the Supplementary Information for further discussion).

The error in the electron affinity of the gold atom is almost 0.3 eV when compared to the experimental value, but it is in good agreement with accurate R-CCSD(T) calculations. In fact, recent calculations by Pasteka and coworkers^58^ show that coupled cluster calculations with up to quintuple excitations and including Breit and QED interactions are needed to obtain an accurate electron affinity. The error in the electron affinity is the only sizable one, and fortunately, it is mostly systematic for all cluster sizes and does not affect the quality of the PES/EDOS comparison. If we correct for that systematic error, the theoretical VDE values (provided in Supplementary Table 4) oscillate about the experimental ones with an additional random error smaller than 0.05 eV. Moreover, the systematic error in the VDE does not affect the chemical bonding, as both structure and dissociation energy are very well described. To demonstrate the systematic nature of this error, we have calculated the vDE and aEA values by taking the difference between the theoretical binding energies of the anionic and the neutral cluster and adding the experimental electron affinity of the atom instead of the theoretical one. The results (shown in Table 1) are then closer to experiment.

Both the benchmark calculations shown in Table 1 and the results shown in the main text confirm that a hybrid functional and an explicit account of dispersion interactions and spin-orbit effects are essential to obtain accurate thermochemistry and electronic properties.

Notice that the D3 correction here employed is transferable across different size regimes, mainly because it has a physically vested functional form and is coupled to an exchange functional (PBE0) that shows almost no spurious dispersion attraction. We have checked that other functionals do not locate the right GM for all sizes, only PBE0-SO-D3 does.

It is also fortunate that for gold, an atom-centered parameterized dispersion model already provides sufficient accuracy, so that there is no need for more expensive non-local vdW functionals. The reason is that the aurophilic dispersion attraction between gold atoms is mostly due to the polarizable, but relatively well localized, 5d orbitals^6^.

## Supplementary information

Supplementary Information

Description of Additional Supplementary Information

Supplementary Data 1

Transparent Peer Review file

## Source data

Source Data

## Data Availability

Coordinates of all cluster structures presented are provided with this paper in Supplementary Data 1. Source data are provided with this paper.
