# Supplementary material for: Structural motifs of gold cluster anions with 17 to 69 atoms
Source: Nat Commun. 2026 Apr 10;17:5099. doi: 10.1038/s41467-026-71649-9 (PMC13246909; doi:10.1038/s41467-026-71649-9)
Supplement: Supplementary file 1 — Supplementary Information [file 41467_2026_71649_MOESM1_ESM.pdf]

Supplementary Information for Structural motifs of gold cluster anions with 17-69 atoms

Andrés Aguado[1*], Pablo Álvarez-Zapatero[1], Oleg Kostko[2,3], Bernd von Issendorff[2,3*]

[1*]Departamento de Física Teórica, Atómica y Óptica, University of Valladolid, Valladolid, 47071, Spain.
[2]Physikalisches Institut, Universität Freiburg, H.-Herder-Str. 3, Freiburg, D-79104, Germany.
[3]Freiburg Materials Research Center, Universität Freiburg, Stefan-Meier-Str. 21, Freiburg, D-79104, Germany.

*Corresponding author(s). E-mail(s): aguado@metodos.fam.cie.uva.es; bernd.von.issendorff@uni-freiburg.de;

Suppl. Note 1. Additional Technical Details of the Calculations

Suppl. Note 1.1. Global optimization runs

The Gupta potential, when expressed in reduced units, depends on two parameters (λ and χ, see main text), which must be provided by the user. We have previously presented a detailed protocol for finding the most appropriate λ and χ parameters for a given metal(1; 2). In those works, we also recommended to use a variety of potentials in a local neighborhood of the optimal potential in order to enhance structural diversity in the data pool wherefrom the DFT optimizations will be later started. For gold clusters, we employ several potentials with λ values in the range $0.45 - 0.49$, and four different values of χ between a minimum $\chi_m = 2$ and a maximum of $\chi_M = 100$, in order to sample structural families with different average coordination numbers and different degrees of bond strain.

The obvious reason for using an empirical potential is to perform a statistically exhaustive exploration of the energy landscape. For each cluster size, we can conduct

between 5 and 10 independent basin hopping (BH) runs, each several million steps long, at a low computational cost. The separate BH runs employ slightly different values for the simulation temperature, for the size of the steps used when moving from one point to the next on the potential energy surface, or for the annealing schedules in those BH runs where the temperature is gradually decreased. By combining the more stable structures from the different runs, we save an initial data pool containing around 4000 structures for each cluster size. After removing duplicates, we sort the remaining structures according to their energy and point group symmetry. Around 400 isomers are then selected from the data pool for further consideration. The detailed selection protocol is the following one: isomers with a point group of order $h \geq 3$ are automatically selected for reoptimization irrespective of their energy ranking in the pool, the rational for this decision being that high-symmetry structures usually identify different structural families and so this procedure enhances diversity. Next, we select the 80-100 most stable structures having point groups with $h = 2$ (C_2, C_s and C_i groups). Finally, the list is filled with the more stable (and more numerous) C_1 structures.

The global optimization runs performed with the neural network potential follow essentially the same protocol, except that the number of separate BH runs was typically smaller. In practical terms, we stopped these simulations once the correct GM structure (as judged by the quality of the EDOS/PES match) was identified. Only for Au_{41}^-, despite several BH runs with both Gupta and NN potentials, the correct GM structure remains unassigned.

Suppl. Note 1.2. Local optimizations and DFT calculations.

As explained in the main text, the selected isomers are locally reoptimized at the PBEsol level of theory(3). At this stage, one can assume, as a working hypothesis, that the relevant structural families have been identified; with only 400 structures in the pool, however, it is possible that the most stable structure within each family has not yet been located. So, once the selected isomers are ranked by their PBEsol energies, we choose to test some additional structures directly on the PBEsol energy surface. The new Au_N^- trial structures are manually generated, either by adding one atom to the more stable isomers of Au_{N-1}^-, removing atoms with low coordination from Au_{N+1}^-, or moving surface atoms of Au_N^- to a different surface site. In the end, this increases the total number of isomers optimized at the PBEsol level by around 100 for each size. Even if these additional structures are generated manually, all of them are based on small modifications within each structural family identified by the unbiased search, so we would like to emphasize that the unbiased empirical potential search is essential for the success of the method.

The PBEsol structural optimizations have been performed with the SIESTA code(4), with each gold atom contributing 11 electrons to the active valence density and core electrons described with norm-conserving Troullier-Martins pseudopotentials(5) and non-linear core corrections(6). The basis set of localized orbitals, explicitly optimized by us in the development of this project, is of triple-zeta

quality plus two additional polarization basis functions per each of the four angular momentum channels included in the basis set (i.e. up to $f-$like basis functions with $l = 3$). The clusters are placed in a large cubic supercell of side length 30 Å. Equilibrium cluster geometries are obtained from unconstrained conjugate-gradients minimization with a force tolerance of 0.01 eV/Å.

The single-point PBE0-D3 and PBE0-SO-D3 calculations, performed on the PBEsol optimal geometry, have been done with the plane-wave code VASP(7; 8). Core electrons are described through the projector-augmented-wave (PAW) approach, employing the latest PAW recommended for gold in the VASP distribution, "accurate" precision settings (plane wave cutoff of 230 eV), and a sufficiently large cubic supercell of side length 30 Å. Dipolar and quadrupolar electrostatic corrections along the three cartesian directions, needed for charged systems in a supercell approach(9), are appropriately included in both SIESTA and VASP calculations. The corrections are applied to the energy, forces and potential.

The large number of structural optimizations with the PBEsol functional were performed with SIESTA (a localized basis set code) for computational efficiency reasons. We have explicitly checked that SIESTA and VASP codes agree at the PBEsol level for a few test sizes. Specifically, we have performed VASP optimizations at the PBEsol level for the global minimum structure of clusters with 21-26 atoms. For the optimal PBEsol-SIESTA geometry (SIESTA forces below 0.01 eV/Å), the PBEsol-VASP forces are always below 0.03 eV/Å. Upon PBEsol-VASP reoptimization, interatomic distances are modified by less than 0.01 Å. These tiny modifications have negligible effect on the electronic eigenvalue spectrum, the quantity of interest in our study.

Suppl. Note 1.3. Accuracy of the single-point calculations performed at the PBEsol optimal geometry

The precision of the single-point approximation was explicitly assessed for a few test sizes by performing PBE0-D3 geometry optimizations and checking that PBEsol and PBE0-D3 optimal geometries hardly differ from each other. More in detail, we performed explicit optimizations at the PBE0-D3 level for the GM structures and competitive isomers of all clusters with $N = 17 - 26$ atoms, and also for size $N = 64$ in order to confirm the uniform validity of the approximation across the wide size range considered in this study. Additionally, we performed a full geometry optimization of Au_{19}^- at the PBE0-SO-D3 level in order to quantify the additional geometry relaxation induced by the spin-orbit interaction. The results of these tests are shown in Supplementary Table 1 and Supplementary Figure 1.

As demonstrated in Supplementary Table 1, we found differences in the bond lengths smaller than about 0.01-0.02 Å when comparing PBEsol and PBE0-D3 optimal geometries. In particular, PBEsol systematically provides distances a bit expanded as compared to PBE0-D3. Spin-orbit effects induce an additional contraction of bond lengths of less than 0.01 Å. The difference between PBEsol and PBE0-D3 remains small for larger clusters such as Au_{64}^- and even for the bulk (PBEsol is in fact closer to experiment than PBE0-D3 with respect to the lattice constant of the crystal).

Supplementary Table 1: Comparison of PBEsol, PBE0-D3 and PBE0-SO-D3 optimal geometries, for a representative set of clusters and the bulk crystal. For each system, we show the shortest, longest and average ($< R >$) bond lengths, and the root-mean-squared deviation of bond lengths with respect to the average value ($\Delta(R)$).

Cluster	Functional	R_{shortest}	R_{longest}	$< R >$	$\Delta(R)$
Au_{17}^-	PBEsol	2.683	2.892	2.764	0.052
	PBE0-D3	2.678	2.887	2.760	0.054
Au_{18}^-	PBEsol	2.676	3.244	2.774	0.080
	PBE0-D3	2.671	3.236	2.766	0.083
Au_{19}^-	PBEsol	2.662	3.367	2.834	0.160
	PBE0-D3	2.656	3.360	2.828	0.164
	PBE0-SO-D3	2.650	3.353	2.821	0.162
Au_{20}^-	PBEsol	2.673	3.067	2.811	0.109
	PBE0-D3	2.665	3.056	2.802	0.113
Au_{22}^-	PBEsol	2.678	3.132	2.782	0.073
	PBE0-D3	2.669	3.124	2.773	0.076
Au_{26}^-	PBEsol	2.676	3.036	2.802	0.081
	PBE0-D3	2.665	3.029	2.800	0.086
Au_{64}^-	PBEsol	2.703	3.591	2.866	0.113
	PBE0-D3	2.697	3.581	2.853	0.114
Bulk	PBEsol			2.879	
	PBE0-D3			2.863	

Maybe the best demonstration that PBEsol geometry is more than enough for the purpose of obtaining a convincing structure assignment based on the quality of the EDOS/PES match is offered by the results in Supplementary Figure 1. It compares the EDOS of Au_{64}^- calculated at the PBE0-SO-D3 level for the relaxed PBEsol and PBE0-D3 geometries: the two curves are so similar that they are visually undistinguishable on the scale of Fig. 2 of the main text.

These test calculations explicitly demonstrate that PBEsol geometries have quantitative accuracy for the purposes of this paper. Only for the special case of Au_{47}^- a more accurate optimization than the one provided by PBEsol was needed to match the PES. As explained in the main text, this particular size exhibits near-degeneracy of two spin isomers at the PBE0-D3 level: a spin triplet with perfect D_{3h} symmetry and a singlet state that undergoes a Jahn-Teller distortion to C_{2v} symmetry. It so happens that the EDOS is extremely sensitive to deformation along the JT coordinate. In order to match the experimental PES with sufficient accuracy as shown in Fig. 2 of the main text, we were forced to perform structural optimizations at the PBE0-SO-D3 level, i.e. with explicit spin-orbit effects included, notwithstanding that these optimizations are very computer intensive. Size $N = 47$ therefore needs a different computational protocol than all other clusters studied here.

As a consequence of the very accurate geometries provided by PBEsol, the relative stabilities of isomers calculated at PBE0-D3/PBEsol level are equally accurate. In cases of near-degeneracy, it is fair to admit that a PBE0-D3 explicit optimization might lead to some re-ordering of the energies of the involved isomers, but our claim is that nearly degenerate isomers at the PBE0-D3/PBEsol level will remain nearly

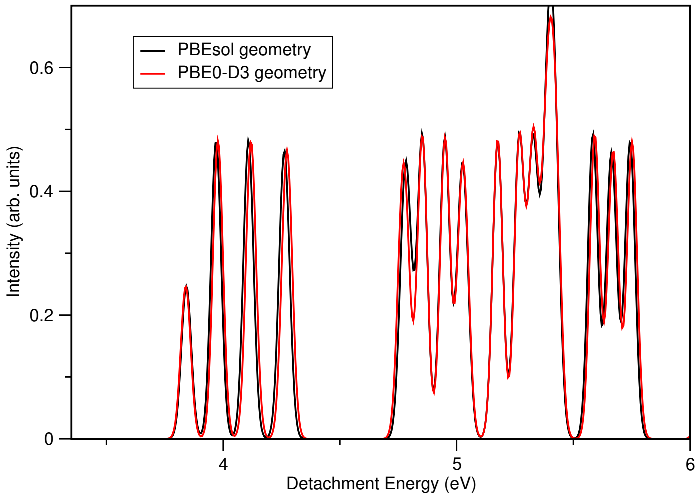

Supplementary Figure 1: The black curve shows the EDOS of the GM structure of Au_{64}^-, obtained from PBE0-SO-D3 calculations on the optimal PBEsol geometry. The red curve shows the EDOS obtained for the optimal PBE0-D3 geometry.

degenerate at the PBE0-D3/PBE0-D3 level of theory. We have performed additional explicit PBE0-D3 optimizations for sizes N=27,32,35 and 39, all displaying competitive or nearly-degenerate isomers. Supplementary Table 2 displays the relevant energy differences at PBE0-D3/PBEsol and PBE0-D3/PBE0-D3 levels of theory. The table demonstrates that the energetic ordering of isomers is not substantially modified upon PBE0-D3 optimization. Only for Au_{39}^-, one of the pyramidal isomers becomes more stable than the polyhedral structure, but by such a tiny amount that the essential conclusion (that the two structural families are nearly degenerate) is not modified.

The thermochemistry (i.e. relative stabilities of isomers) provided by PBEsol/PBEsol is unfortunately not as accurate as the geometry; nevertheless, it is still useful in practical terms, because it helps to select which isomers need to be explicitly evaluated at the PBE0-D3 and PBE0-SO-D3 levels of theory. We have found that the PBE0-SO-D3 total energy difference between two given isomers may differ from its PBEsol counterpart by typical values around 0.1 eV, and have used that observation to select only those isomers whose PBEsol energy is up to about 0.2 eV above the PBEsol GM energy (up to about 0.4 eV for sizes smaller than $N = 26$) for further consideration at PBE0-D3 and PBE0-SO-D3 levels. Supplementary Table 3 reports explicit data on the relative stabilities of different isomers for some representative sizes, comparing PBEsol and PBE0-SO-D3 results, in order to demonstrate the validity of our protocol.

The results clearly show that the PBEsol/PBEsol energetic ordering of isomers is not to be trusted; a PBE0-SO-D3/PBEsol re-evaluation of stabilities is absolutely necessary. Still, the PBEsol energies are reasonable enough to provide a useful guide towards the more stable candidate structures. For many sizes, the PBEsol GM structure in fact coincides with the PBE0-SO-D3 GM structure; and even for those sizes

Supplementary Table 2: Comparison of the relative stabilities of isomers obtained at PBE0-D3/PBEsol and PBE0-D3/PBE0-D3 levels of theory, for a representative set of cluster sizes showing near degeneracy. The different isomers are named with roman numbers (I, II, III, etc.), according to their PBE0-D3/PBEsol stabilities, with (I) being the GM structure, (II) the first structural excitation, etc. Energy differences with respect to the corresponding GM structure are given in eV.

Size	Isomer	PBE0-D3/PBEsol	PBE0-D3/PBE0-D3
$N = 27$	I (D_{6d})	0	0
	II (C_s)	0.070	0.050
$N = 32$	I (D_{3h})	0	0
	II (C_2)	0.001	0.015
	III (C_1)	0.021	0.032
$N = 35$	I (C_3)	0	0
	II (C_1)	0.028	0.017
$N = 39$	I (C_{2v})	0	0.007
	II (C_1)	0.010	0
	III (C_1)	0.011	0.003

for which the two GM structures differ, the true GM structure can usually be found within an energy window of 0.2 eV above the PBEsol minimum energy. Although only a selection of sizes is shown in Supplementary Table 3, the conclusion is valid for all sizes considered in this study.

Suppl. Note 1.4. Vertical detachment energies

As mentioned in the main text, vertical detachment energies (VDE) of Au_N^- anions are calculated through a Δ−SCF calculation, that is, as the total energy difference between the anion and the neutral cluster, both clusters adopting the geometry of the anion. The theoretical VDE values, as well as the magnitude of the energy shifts applied to each size, are given in Supplementary Table 4. The mostly systematic nature of the shift is evident from the table: once that systematic error is corrected, the theoretical VDE values reproduce the size dependence of the experimental VDE values within an error bar smaller than 0.05 eV.

The error in the calculated EA of about 0.3 eV is already present for the gold atom. As mentioned in the main text, this property of the gold atom has been thoroughly studied in a work by Pasteka et al.(10). The authors reached agreement with the experimental EA value on a meV level, providing a definitive resolution for a long-standing problem. Their work demonstrates that, in order to reach such a precision, (1) an all-electron correlated description is required, in particular correlating the 5d shell with the inner n = 4 shell, and including up to pentuple excitations in the coupled cluster expansion; (2) a four-component relativistic calculation at the Dirac- Coulomb-Hartree-Fock level, including a parameterized description of the finite size of the nucleus (hyperfine effects), is required; and (3) the Breit interaction

and QED effects (self-energy of the electron, Lamb shift operator, vacuum polarization operator, etc.) must be self-consistently included together with the electron correlation effects. These relativistic and inner-shell correlation effects have a direct influence mostly on the core electrons, but indirectly also affect valence properties and the spatial extent of the electron density. This in turn influences the EA value, a quantity that is in principle determined by the long-range asymptotic decay of the XC potential. Applying such precise calculations to clusters of arbitrary size would clearly be prohibitive. What our calculations demonstrate is that the error in the EA of the gold atom seems to be systematically transferred to cluster anions of arbitrary size. We can only speculate that this is due to an incorrect asymptotic tail of the decaying electron density, which should display similar features for the atom and the clusters (all having the same total charge of -1). Any other explanation would be hard to reconcile with the fact that all structural and bonding properties are well described, which indicates that the error must be caused in a region of space that is not important for bonding properties. Future DFT functionals, which better describe the long range tail of the electron density (without changing the bonding properties), might therefore also provide better estimates of the EA.

Supplementary Table 3: Comparison of the relative stabilities of isomers obtained at PBE0-SO-D3 and PBEsol levels of theory, for a representative set of cluster sizes. The different isomers are named with roman numbers (I, II, III, etc.), according to their PBE0-SO-D3 stabilities, with (I) being the GM structure, (II) the first structural excitation, etc. Energy differences with respect to the corresponding GM structure are given in eV. All energies are single-point energies calculated at the optimal PBEsol geometries.

Size	Isomer	PBE0-SO-D3	PBEsol	Size	Isomer	PBE0-SO-D3	PBEsol
Au_{17}^-	I	0.000	0.000	Au_{18}^-	I	0.000	0.000
	II	0.065	0.130		II	0.042	0.038
	III	0.133	0.108		III	0.046	0.051
	IV	0.163	0.096		IV	0.126	0.113
Au_{19}^-	I	0.000	0.000	Au_{20}^-	I	0.000	0.000
	II	0.477	0.356		II	0.430	0.347
	III	0.546	0.406		III	0.445	0.360
					IV	0.463	0.392
Au_{21}^-	I	0.000	0.000	Au_{26}^-	I	0.000	0.000
	II	0.032	0.220		II	0.019	0.014
	III	0.057	0.353		III	0.042	0.085
	IV	0.119	0.211		IV	0.108	0.110
	V	0.127	0.060		V	0.110	0.062
	VI	0.254	0.381		VI	0.125	0.057
Au_{33}^-	I	0.000	0.086	Au_{60}^-	I	0.000	0.099
	II	0.061	0.090		II	0.012	0.000
	III	0.065	0.000		III	0.030	0.059
	IV	0.095	0.099		IV	0.031	0.066
	V	0.096	0.104		V	0.110	0.058
	VI	0.146	0.022		VI	0.136	0.142

Supplementary Table 4: Comparison of experimental and theoretical vertical detachment energies (in eV). The experimental values correspond to the energy of the first (i.e. lowest-energy) main peak in the PES. The theoretical values correspond to the VDE of the structure that matches that peak. This is the GM structure for most sizes but it can be an excited isomer (in the cases of hidden GM structures discussed in the main text). The E_{shift} column shows the shifts applied to the EDOS in Fig. 2 of the main text in order to get the best possible EDOS/PES match. E_{shift} is almost always equal to the difference between experimental and theoretical VDE values. One exception, for example, is Au_{17}^-, where a small offset between the experimental and theoretical position of the lowest binding energy peak leads to a better overall EDOS/PES match (see Fig. 2).

Cluster	Exp.	PBE0-SO-D3	E_{shift}	Cluster	Exp.	PBE0-SO-D3	E_{shift}
Au_{17}^-	4.08	3.75	0.29	Au_{49}^-	4.18	3.88	0.30
Au_{20}^-	2.81	2.55	0.28	Au_{51}^-	4.19	3.87	0.31
Au_{21}^-	3.95	3.66	0.29	Au_{52}^-	4.15	3.86	0.29
Au_{25}^-	4.05	3.75	0.30	Au_{55}^-	4.33	4.05	0.28
Au_{27}^-	4.08	3.80	0.28	Au_{57}^-	4.30	4.03	0.27
Au_{29}^-	4.02	3.72	0.30	Au_{58}^-	3.56	3.29	0.27
Au_{32}^-	4.02	3.72	0.30	Au_{60}^-	3.68	3.39	0.29
Au_{33}^-	4.19	3.87	0.32	Au_{62}^-	3.79	3.50	0.29
Au_{35}^-	4.10	3.80	0.30	Au_{63}^-	3.98	3.68	0.30
Au_{39}^-	4.07	3.76	0.31	Au_{64}^-	3.85	3.56	0.29
Au_{42}^-	3.88	3.62	0.26	Au_{67}^-	4.02	3.73	0.29
Au_{47}^-	3.97	3.67	0.30	Au_{69}^-	4.15	3.87	0.28
Au_{48}^-	3.98	3.69	0.29				

Suppl. Note 2. Additional Details of the Benchmark Calculations

For the benchmark calculations shown in Fig. 1 and Table 1 of the main text, we increased the plane wave cutoff in the VASP calculations to 300 eV in order to get fully converged binding energies. We decided to take the Au_{19}^- cluster anion as a test system to analyze the performance of several exchange-correlation functionals in reproducing the corresponding photoemission spectrum (the observable of interest in this study). The cluster structure was relaxed using PBEsol; all further calculations were single-point ones for this geometry. Specifically, we tried all of the GGA, meta-GGA and hybrid functionals explicitly included and documented in the VASP distribution (a total of 33 different functionals in the version 5.4.1 of VASP). Concerning hybrid functionals, we added variations of the PBE0 functional to our test set, obtained by modifying the amount of non-local exchange.

Figure 1 of the main text demonstrates that only a hybrid functional with an explicit account of spin-orbit effects can produce an EDOS that accurately matches the PES. Here we show the complete set of benchmark calculations. Supplementary Figure 2 displays the results obtained from semi-local functionals (both GGA and meta-GGA). For each functional, we rigidly shifted the eigenvalue spectrum so that

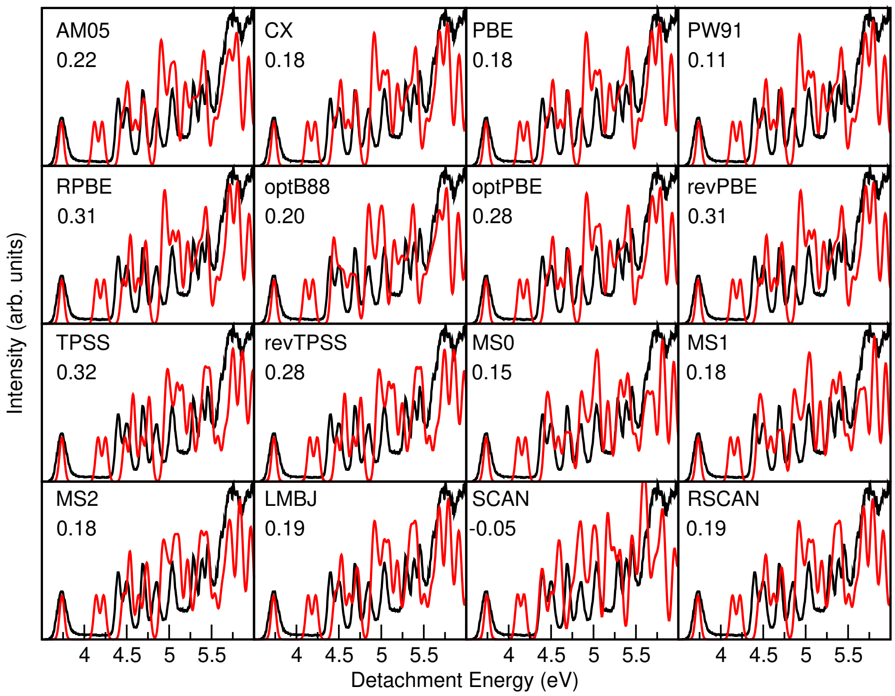

Supplementary Figure 2: Performance of semi-local functionals in reproducing photoemission spectra: the black curves show the experimental PES of Au_{19}^{-}, the red curves show the corresponding EDOS, as obtained from theoretical calculations using GGA (first and second rows) and meta-GGA (third and fourth rows) functionals. The functional acronym, as detailed in the VASP documentation, and the global shift applied to the theoretical EDOS (in eV), are included in each subgraph. In all calculations spin-orbit effects have been taken into account; the cluster structure was relaxed at the PBEsol level.

the HOMO coincides with the experimental vertical detachment energy. This figure clearly illustrates that the different GGA functionals produce very similar densities of states, which, however, do not match the experimental results at all, irrespective of their different parameterizations. The much more computer-intensive meta-GGA functionals barely improve the situation, if at all, and once more produce very similar EDOS curves even if they are parameterized in different ways. We conclude that semi-local functionals can not produce a realistic density of states for gold clusters. Nevertheless, it is interesting to notice that the SCAN meta-GGA approximation is the functional that best matches the experimental VDE value (the global shift of the EDOS being very small for this functional).

Supplementary Figure 3 displays the corresponding results obtained using hybrid functionals. Just at a glance it is obvious that hybrid calculations under a generalized Kohn-Sham (GKS) scheme perform much better; most of the hybrid functionals produce acceptable results, but only the original PBE0 functional leads to the most

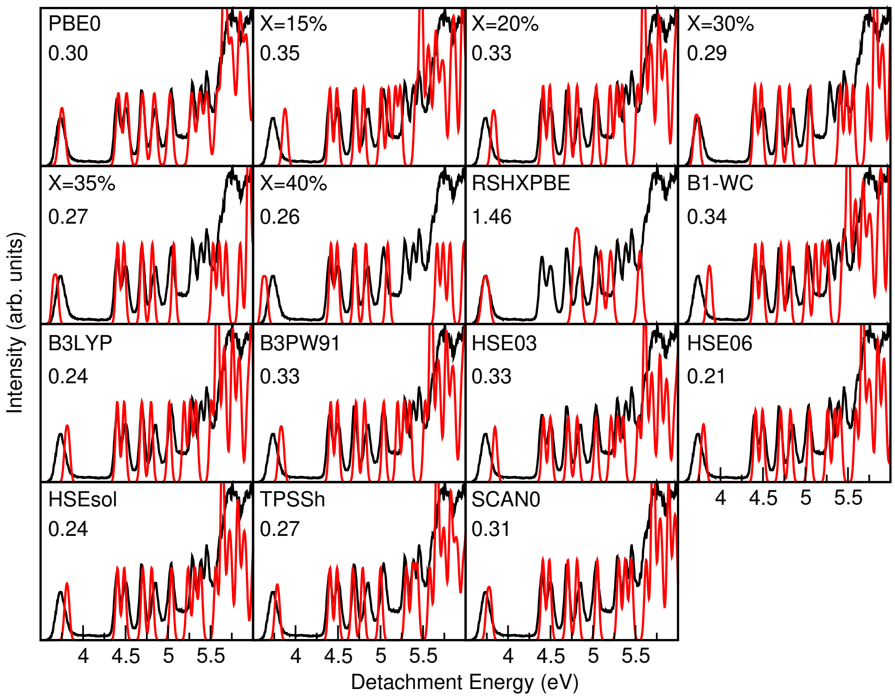

Supplementary Figure 3: Performance of hybrid functionals (including spin-orbit coupling) in reproducing photoemission spectra. X gives the amount of nonlocal exchange used in combination with the PBE functional. Rest of the caption as in Supplementary Figure 2

accurate match to the experimental PES. Other unscreened hybrids based on GGA, such as B3LYP or B3PW91 perform clearly worse, even if they retain an acceptable level of accuracy. Range-separation (HSE03, HSE06 and HSEsol functionals) does not improve the agreement for this particular observable of gold clusters, even if it slightly reduces the systematic error in the electron affinity (HSE06 and HSEsol functionals). Concerning the meta-GGA hybrids, it is important to emphasize that SCAN0 is the one functional whose performance is closest to PBE0: only the energy gap between the first two peaks is slightly underestimated by SCAN0.

Supplementary Figure 3 shows additional results obtained by modifying the amount of non-local exchange within PBE0, thus deviating from the 25% exchange implemented in the original PBE0. In particular, we tried values of $X = 15\%, 20\%, 30\%, 35\%$ and 40%. Only for these curves, we decided to rigidly shift the different EDOS so that all of them approximately match the second-to-fifth peaks in the PES, just for visualization purposes. Finally, we also tested the RSHXPBE functional, which has full (100%) non-local exchange. These results not only show that non-local exchange effects are important in order to obtain an accurate EDOS, but also that it is indispensable to add the right amount X_{opt} of non-local exchange, which in fact

coincides with the 25% used in the original PBE0 hybrid. For $X > X_{\rm opt}$, the molecular orbitals with a more dominant $d-$character are pushed to higher binding energies, spoiling the agreement with experiment (in the extreme case of $X = 100\%$, most of the peaks in the EDOS are no longer seen in the energy window shown in the figure); for $X < X_{\rm opt}$, exactly the opposite happens: the subset of three peaks located just before the rise of the "$d-$band" shifts to lower energies and, for $X = 15\%$, even overlaps with the next lower binding energy peak. This is the most noticeable effect, but the gap between the first and the second peak is also affected by the specific amount of non-local exchange: the gap is too narrow for $X < X_{\rm opt}$ and too wide for $X > X_{\rm opt}$.

Once we identified the optimal level of theory for our purposes, we performed the benchmark assessment shown in Table 1 of the main text, which was done in order to check whether PBE0 provides the right EDOS for the right reasons. The right functional should not only yield a good EDOS/PES fit, but also provide accurate structural, electronic and energetic properties, and this uniformly from the isolated atom to the bulk crystal limit. The calculations were performed with and without explicit spin-orbit effects, and with and without the D3 dispersion correction, in order to assess the relative importance of these two effects on the structural, energetic, vibrational and electronic properties, as displayed in Table 1 of the main text. The DFT calculation results were benchmarked against both experimental results and very high-quality R-CCSD(T) small-core calculations.

The whole set of results demonstrates that the original PBE0 hybrid, with explicit spin-orbit and dispersion corrections, provides very accurate results for all of the different properties. Although this conclusion was initially extracted from the benchmark analysis only (Table 1 and Fig. 1 of the main text), it is crucial to emphasize that it was strongly corroborated by the quality of the results for the gold cluster anions with $N = 17 - 70$ atoms, examples of which are shown in Fig. 2 of the main text.

In the following, we provide additional details of the benchmark calculations. We have explicitly optimized all the structures of gold clusters with $N = 3 - 8$ atoms as reported in a previous extensive benchmark study by Baek *et al.*(11). In Table 1 of the main text, we report that the mean absolute error (MAE) of atomic coordinates as compared to their R-CCSD(T) results(11) is below 1% for all clusters in the size range $N = 3-8$. In Supplementary Fig. 4, we additionally show that the relative stabilities of the different competitive isomers compare equally well with the R-CCSD(T) results. In particular, a systematic trend is observed as a function of basis set size in the R-CCSD(T) calculations: three-dimensional structures are always stabilized (with respect to the planar structures) when the basis set is augmented from TZ to QZ size. Exactly the same trend is observed in the VASP calculations when the plane-wave cutoff is augmented from 230 eV (default value in "accurate" VASP settings) to 300 eV.

Although the agreement with experiment is very good regarding structure and cohesion, the present situation is less satisfying if we consider the ionization potentials. Despite the fact that the available experimental values also differ quite a bit, there still seems to be a systematic error in the calculated values. This was also demonstrated by the benchmark study by Baek *et al.*(11), who tested 44 different DFT functionals as well as R-CCSD(T) calculations. None of their calculations could reproduce the

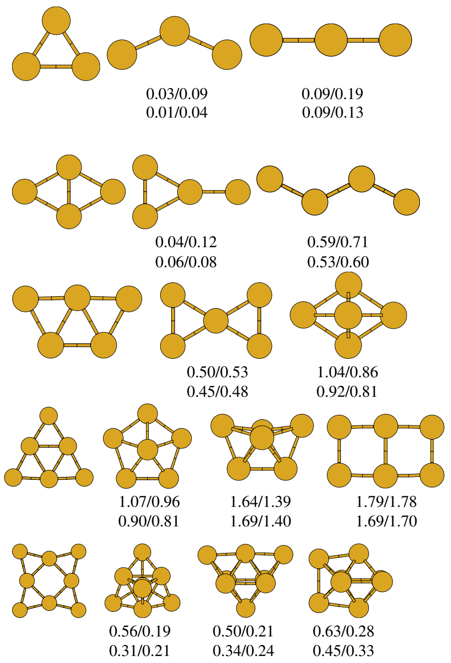

Supplementary Figure 4: Structures of neutral Au_N clusters with $N = 3, 4, 5, 6, 8$. Numbers below each isomer are total energy differences with respect to the GM structure. First row: CCSD(T) calculations with a basis set of TZ/QZ quality(11); second row: our PBE0-SO-D3 results with plane wave cutoff of 230/300 eV.

experimental vIP values as determined by Jackschadt *et al.*(12) using electron impact ionization. The MAE of the theoretical vIP values was as large as 0.8 eV when compared to experiment for both DFT and R-CCSD(T), while the agreement between DFT and R-CCSD(T) was much better. Our results in Table 1 of the main text confirm that PBE0-SO-D3 results for the vIP are in very good agreement with CCSD(T) results, which we take as the accuracy standard for this quantity. It is possible that the electron impact technique is a bit problematic for metal clusters, as photoionization measurements resulted in rather different values for the ionization potentials(13). So, while theory can still be improved, there certainly also remains a need for more precise experimental data.

Suppl. Note 3. Additional Structural Data

In this section, we provide the structures that were not explicitly included in Fig. 3 of the main text for space reasons. In particular, Fig. 2 of the main text includes the EDOS of all isomers that contribute to the experimental PES, but some of these isomers were not shown in Fig. 3. These isomers are shown in the Supplementary Fig.

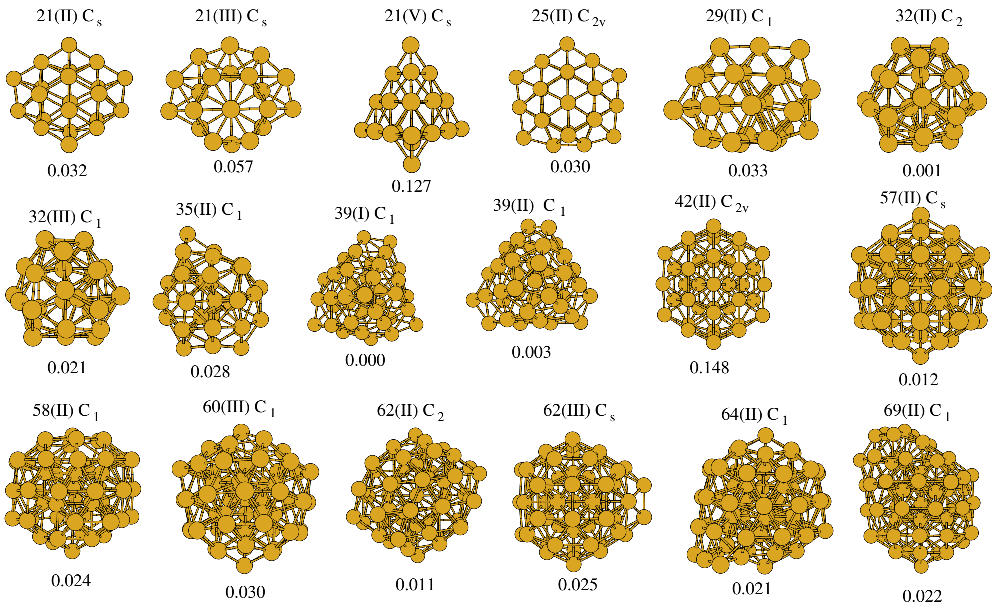

Supplementary Figure 5: Structures of competitive isomers that contribute to the experimental PES signals. The cluster size, isomer number and approximate point group symmetry are given above each image; the energy difference with respect to the GM structure is given, in eV, below each image.

5. Here we briefly repeat the description of some of these structures. All the main peaks in the PES of Au_{21}^- (see Fig. 2 of the main text) are perfectly reproduced by the EDOS of the GM structure. Isomer II is another member of the hexagonal prismatic structural family, 0.03 eV less stable than the GM structure, and explains two weaker features in the PES. Isomer III, based on a hexagonal antiprism, might contribute as well to the PES, but all the peaks in its EDOS are hidden by signals coming from the two more stable structures (the same occurs for isomer IV, not shown explicitly in Fig. 2). Finally, the extremely weak feature detected in the PES at low energies can only be matched by isomer V, a 20-atom pyramid with an external adatom. This demonstrates the presence of a minor population of pyramidal isomers in the experimental cluster beam for this particular size.

Concerning the remaining isomers shown in Supplementary Fig. 5, we briefly discuss only the most interesting examples. Isomer II of Au_{25}^- is still a member of the hexagonal prismatic structural family. In this case the hexagonal prism is almost fully saturated with adatoms. It is only 0.03 eV less stable than the GM structure and clearly contributes to the PES (Fig. 2 of the main text). This is therefore the last size for which hexagonal prisms are still competitive, and also the size for which a structure with one internal atom becomes stable. Isomers II and III of Au_{32}^- are shown in Supplementary Fig. 5 with the appropriate orientation to demonstrate that their external shell of atoms is exactly the same (except for slight distortions) as that

of the GM structure. They differ from the GM structure just in the precise orientation of the triangular core with respect to the shell, and are therefore almost exactly degenerate. As a consequence, the C_1 distortion (isomer III) is the most abundant one, due to its entropic advantage. For Au_{39}^-, isomers I and II are chiral pyramids that are nearly degenerate with the C_{2v} structure, and that dominate the PES again due to their entropic advantage. The isomer II of Au_{42}^- is a perfect icosahedral fragment, obtained by removing atoms from a 55-atom Mackay icosahedron in such a way that only four internal atoms remain in the structure. The remaining isomers shown in Supplementary Fig. 5 are all members of the frustrated icosahedral family.

Suppl. Note 3.1. Common Neighbor Analysis of Selected Structures

In this subsection we offer a common neighbor analysis (CNA)(14) of a few structures selected from the frustrated icosahedral family. This type of analysis may help to assess in a more quantitative way both the degree of icosahedral ordering present in what we call "frustrated icosahedra" and the nature of the local defects that produce such a frustration.

As explained in the main text, we identified frustrated icosahedra by a detailed visual inspection of the structures, i.e. by assessing that the core of the cluster is an icosahedral fragment, and that the shell contains a significant amount of the pentagonal 16-atom caps that characterize the surface facets of a Mackay icosahedron in the size range around 55 atoms. We also included defects that are typically seen during icosahedral growth, such as the rosette defects, as part of our definition of "frustrated icosahedral" family. These purely visual descriptors clearly underline the structural trends that we wanted to emphasize.

CNA descriptors can certainly provide a more quantitative measure of the degree of icosahedral order. CNA assigns three different indicators (n, k, l) to each bond in the cluster, i.e. to each pair of atoms, A and B, connected by a bond. n is the number of atoms that are simultaneously nearest neighbors of both A and B, i.e., the number of "common neighbors" (CN); k is then the number of bonds between those CN's; finally, l is the length of the longest uninterrupted chain of bonds that can be formed between those CNs. The obvious problem with a traditional implementation of the CNA method(14; 15) is that a "bond" is defined by a single external parameter: the cutoff radius. Therefore, it provides a robust analysis only for nanoparticles that have a wide gap separating nearest from next-nearest neighbor bond lengths, as it occurs for example in a perfect Mackay icosahedron (discussed below). But the gold nanoparticles studied here display some degree of surface disorder, due to the structural frustration (defects, local relaxations, etc.), which results in a less clear separation between first and second neighbors. Therefore, a traditional CNA can fail in displaying the structural trends that we clearly identified through visual inspection. Therefore, we have decided to use a home-built version of the so-called interval-CNA (i-CNA)(16), where a finite interval of cutoff distances is analyzed instead. In detail, we have analyzed cutoff values between 3.4 and 3.8 Å, and have chosen the most appropriate signatures (those that remain stable in a wider range of the tested cutoffs) for the correct identification of the local environment around each selected bond. The i-CNA method is

Supplementary Table 5: Common Neighbor Analysis for Ih_{55} (a perfect Mackay icosahedron) and for Au_N^- cluster anions with $N = 55, 57, 58, 62$ and 69. For each cluster, the first row displays the total number of bonds involving core atoms N_{core} (the sum of core-core and core-shell bonds), the total number of shell-shell bonds N_{shell}, and the total number of bonds with signature $n = 2 - 6$; the second row displays the percentage values of N_{core} and N_{shell} with respect to the total number of bonds, and the percentage values of bonds with a given n value (with respect to N_{core} for $n = 4, 5, 6$, and wrt N_{shell} for $n = 2, 3$). The very few bonds with $n = 6$ are treated together with those having $n = 5$ in the statistical analysis; finally, the third row displays the number of bonds with a given (kl) signature for each value of n.

Cluster	N_{core}	N_{shell}	$n = 5 + n = 6$	$n = 4$	$n = 3$	$n = 2$
Ih_{55}	114	120	24	90	120	0
	48.7%	51.3%	21%	79%	100%	0%
			(555): all	(422): all	(322): 60	
					(311): 60	
Au_{55}^-	88	134	19	69	108	26
	39.6%	60.4%	21.6%	78.4%	80.6%	19.4%
			(555): 8	(422): 50	(322): 44	(211): 9
			(543): 11	(433): 11	(311): 61	(200): 17
				(421): 8	(300): 3	
Au_{57}^-	95	140	20	75	118	22
	40.4%	59.6%	21.1%	78.9%	84.3%	15.7%
			(555): 13	(422): 60	(322): 49	(211): 10
			(543): 7	(433): 7	(311): 64	(200): 12
				(421): 3	(300): 5	
Au_{58}^-	117	123	27	90	96	27
	48.75%	51.25%	23.1%	76.9%	78%	22%
			(555): 18	(422): 72	(322): 44	(211): 15
			(543): 9	(433): 6	(311): 49	(200): 12
				(421): 12	(300): 3	
Au_{62}^-	117	144	24	93	135	9
	44.8%	55.2%	20.5%	79.5%	93.7%	6.3%
			(666): 3	(422): 81	(322): 69	(200): all
			(555): 21	(433): 9	(311): 66	
			(543): none	(421): 3		
Au_{69}^-	133	158	33	100	131	27
	45.7%	54.3%	24.8%	75.2%	83%	17%
			(666): 1	(422): 66	(322): 57	(211): 9
			(555): 18	(433): 25	(311): 74	(200): 18
			(543): 14	(421): 9		

much more robust because it correctly identifies that positive and negative disclination defects (such as rosettes and square defects, for example) have different stability intervals that cannot be captured with a single cutoff.

The results of our CNA are summarized in Supplementary Table 5. We consider the perfect Mackay icosahedron with 55 atoms (Ih_{55}) as a reference structure, i.e. we first calculate the CNA signatures of Ih_{55} and then compare the CNA signatures of selected frustrated icosahedra to that reference. The Ih_{55} cluster has a total of 234

bonds: 24 bonds with 5 CNs ($n = 5$); 90 bonds with 4 CNs ($n = 4$), and the remaining 120 bonds with $n = 3$. There are no bonds with $n = 6$ in a perfect Mackay icosahedron. All bonds with $n = 5$ are (555) bonds, and correspond to the 12 bonds formed between the central atom and its 12 nearest neighbors, plus 12 additional bonds between each pentagonal vertex in the shell and the atom immediately below; therefore, (555) bonds are radial bonds. All bonds with $n = 4$ are of the (422) type: they include the 30 tangential bonds between pairs of outer core atoms, plus 60 radial core-shell bonds, so all of them involve the core atoms. Finally, the bonds between pairs of surface atoms have $n = 3$: 60 of them are of the (322) type and involve the vertex atoms, and the remaining 60 bonds are of the (311) type and involve only the ridge atoms. In summary, for Mackay icosahedra of this size, around 10,2% of the bonds have $n = 5$, 38,5% of bonds have $n = 4$, and 51,3% of bonds have $n = 3$. These proportions can be taken as a gross quantitative measure of compact icosahedral ordering for clusters with around 55 atoms - of these the most significant one is the presence of a non-negligible proportion of bonds with five CN's. The narrow distribution of the two remaining indexes (k, l) adds to that simple description and provides a finer measure of what can be considered "perfect" icosahedral order.

In order to compare the CNA signatures of frustrated icosahedra to those of the Ih_{55} reference in a fair and meaningful way, we have to take into account the fact that gold clusters are less compactly packed than perfect icosahedral nanoparticles, i.e. the number of core atoms is smaller than in a compact Mackay icosahedron (Fig. 4 of the main text). For example, Au_{55} has only 10 core atoms, while a Mackay icosahedron has 13 core atoms. Therefore, the proportion of bonds with 5 or 4 CN's (bonds involving core atoms) will be systematically reduced in frustrated icosahedral gold clusters as compared to compact Mackay icosahedra. As a direct consequence, the proportion of bonds with $n = 3$ (the bonds between pairs of surface atoms) will be systematically enlarged as compared to a compact Mackay icosahedron. Therefore, when comparing with the perfect 55-atom icosahedron, Supplementary Table 5 displays the percentages of bonds with $n = 4, 5$ with respect to the total number of bonds involving at least one core atom (and the percentages of bonds with $n \leq 3$ with respect to the total number of surface-surface bonds).

On general grounds, we expect that bonds with a given value of n will display a wider distribution of possible (kl) values in the frustrated icosahedra, because these two last signatures are much more sensitive to the presence of structural defects such as the core bubbles or the square facets in the cluster shell observed in our cluster structures. Also, while in the perfect Mackay icosahedron all bonds between shell atoms have $n = 3$, in frustrated icosahedra we expect to observe some shell-shell bonds with $n = 2$, related with the square defects that locally reduce packing density within the shell. The results in Supplementary Table 5 perfectly conform to these expectations: the first CNA signature (n) serves to quantify the structures as "icosahedral", because the relative percentages of bonds with $n = 5$, $n = 4$ and $n = 3$ are very close to the expected ones for an icosahedron, once we correct them for the fact that gold clusters are less densely packed. The distribution of the remaining two signatures (kl), for each value of n, quantifies then the degree of "frustration".

More in detail, we have observed that not all bonds with $n = 5$ are of the (555) type, but there is a non negligible proportion of (543) bonds, which quantifies a distortion where the local environment of those bonds is "less compact" as compared to the perfectly icosahedral (555) bonds. Similarly, most bonds with $n = 4$ are of the (422) type as in a perfect icosahedron, but with a non-negligible proportion of (433) bonds, which in this case identify a more compact local environment as compared to (422) bonds, and a generally smaller amount of (421) bonds. The surface-surface bonds with $n = 3$ continue to be mostly (322) and (311) bonds in roughly 50/50 proportion. All in all, the i-CNA corroborates that the structures are predominantly icosahedral, and help in characterizing the origin of frustration by identifying the new types of bond that result from that frustration.

As a final comment of only secondary importance, we identify a few bonds with $n = 6$ in our gold clusters, associated with the rosette defects. The local environment of these bonds is a hexagonal bi-pyramid. These bonds obviously do not occur in the perfect Mackay icosahedron with 55 atoms, but are easily observed in icosahedral clusters such as Na_{56} or Na_{57}, for example(17). We therefore consider, in practical terms, those bonds as characteristic of icosahedral growth in cluster systems, and consider them together with the $n = 5$ bonds in the statistical analysis shown in Supplementary Table 5.

Suppl. Note 4. Structure determination: quantitative assessment of the EDOS/PES agreement

Photoelectron spectra are usually very sensitive to cluster structure and therefore can be used for structure assignment. In Fig. 2 of the main text we have demonstrated that the EDOS of the putative GM structures are in very good agreement with the measured PES, accept for the cases of "hidden" minima as discussed. This structural assignment is only reliable if other structural isomers yield a worse agreement between the EDOS and the PES. In Supplementary Fig. 6 we show Fig. 2 of the main text again, but now with the EDOS of the next higher lying isomers. In detail, we select three additional isomers for each cluster size, specifically those energetically directly above the ones that contribute to the PES, i.e. to the ones already shown in Fig. 2 of the main text. For example, for Au_{17}^-, for which only the GM structure contributes to the PES, we display the EDOS of isomers II, III and IV; for Au_{21}^-, for which even the fifth isomer seems to slightly contribute to the PES, we display the EDOS of isomers VI, VII and VIII. A detailed description of the geometrical structures of all low lying isomers in the size range N=17-70 will be presented in forthcoming publications.

The figure confirms that the EDOS of excited isomers generally cannot match the PES. Some of these excited isomers do not belong to the same structural family as the GM structure, and therefore their EDOS are usually significantly different. But even in cases where an isomer belongs to the same structural family as the GM, the EDOS usually is still sufficiently different so that we can rule out the presence of these isomers in the experiment. Very clear examples of this situation are sizes 17,20,21,42,47,51,58 or 63.

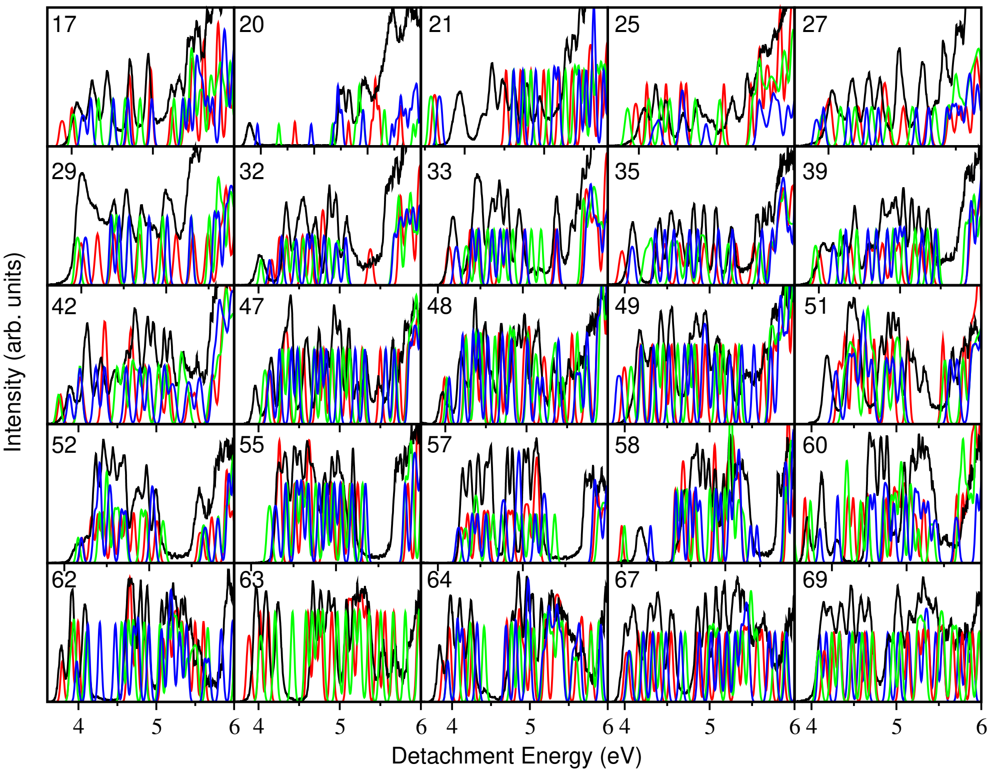

Supplementary Figure 6: Measured photoelectron spectra in comparison to the EDOS of the three most stable isomers not shown in Fig. 2 of the main text. Red, green and blue lines indicate decreasing stability of the isomers. For Au_{63}^- PBE-SO-D3 calculations were only performed for two additional isomers.

In order not to rely too much on visual inspection, but to really quantify the agreement between the EDOS and the PES, we have used a simple fitting approach. In detail, we have fitted the experimental PES by using a parametrized EDOS:

$$f(E) = A \sum_{n=1}^{N_{KS}} \exp\left(-\frac{(E - E_n - E_o)^2}{2\sigma^2}\right).$$

Here E_n are the Kohn-Sham energies from the PBE0-SO-D3 calculations, N_{KS} is the number of Kohn-Sham levels in the region of interest, σ is a fixed width parameter (set to 30 meV in most cases), A is the overall amplitude and E_o is an offset energy accounting for the slight shift between EDOS and PES. A and E_o were used as fitting parameters, optimizing the rms-deviation between the measured PES and the fitting function. In some cases we have used superpositions of the fitting functions of two isomers, in this case employing three fitting parameters, the amplitudes of the two fitting functions and the offset energy used for both of them. For the fitting, the energy

region of the $d-$band has been excluded, as the DFT calculations systematically overestimate the binding energies of these states. The residual error was then calculated employing a standard χ^2 test:

$$\chi^2 = \frac{1}{N_p - 2} \sum_{i=1}^{N_p} \left(I(E_i) - f(E_i) \right)^2 .$$

Here $I(E)$ is the measured spectrum, consisting of N_p points. Such a fit is very crude, as it neither takes into account the state dependence of the photoemission cross sections, nor the possible differences in peak widths due to different final state lifetimes and the amount of vibrational excitation upon photoemission - nevertheless the outcome is unambiguous. Examples of fits are shown in Supplementary Fig. 7. Here the measured spectrum of Au_{48}^- is shown, together with fitting curves corresponding to the four lowest isomers. Visual inspection alone already shows that only the EDOS of the GM (isomer I) can reproduce the measured data; the calculated value of χ^2 corroborates this clearly. In Supplementary Table 6 we list the relative energies and χ^2 values of the lowest isomers for all cluster sizes presented in Fig. 2 of the main text. It can be seen that in almost all cases the EDOS of the putative GM yields the lowest χ^2 values. Exceptions are the cases where the GM is "hidden", as discussed in the main text, or where more than one isomer contributes to the measured spectrum due to energetic degeneracy. In the latter cases a fit assuming the superposition of two isomers yields a significantly better agreement. This result demonstrates the degree of consistency obtained in this study; it is almost always the lowest energy isomer which yields the best EDOS/PES agreement - and in the few exceptional cases there is usually a clear explanation why this is not the case.

Supplementary Table 6: Assessment of the agreement between the measured PES and the calculated EDOS. For all sizes discussed in the main text we list the relative energies (in eV) and χ^2 values of the agreement between PES and EDOS for the lowest lying isomers. Energies have been calculated at the PBE0-SO-D3 level; all structures have been relaxed at the PBEsol level, except for the two lowest isomers of size $N = 47$, which have been relaxed at the PBE0-SO-D3 level, and the three lowest isomers of $N = 39$, which have been relaxed at the PBE0-D3 level (see also Supplementary Table 2). For some of the sizes a superposition of the EDOS of two isomers has been used as well - in this case the relative contributions are given (in parentheses).

Cluster	Isomer	ΔE	χ^2	Cluster	Isomer	ΔE	χ^2	Cluster	Isomer	ΔE	χ^2
Au_{17}^-	I	0.000	0.0171	Au_{35}^-	I	0.000	0.0381	Au_{57}^-	I	0.000	0.017
	II	0.065	0.0564		II	0.028	0.013		II	0.012	0.0185
	III	0.133	0.0431		I+II	(0.21,0.79)	0.0102		III	0.031	0.027
	IV	0.163	0.0493		III	0.043	0.0308		IV	0.451	0.0483
Au_{20}^-	I	0.000	0.0123		IV	0.043	0.0436		V	0.818	0.0742
	II	0.430	0.0172		V	0.050	0.0313	Au_{58}^-	I	0.000	0.0403
	III	0.445	0.0395	Au_{39}^-	I	0.000	0.052		II	0.024	0.0073
	IV	0.463	0.0405		II	0.003	0.0198		I+II	(0.08,0.92)	0.0069
Au_{21}^-	I	0.000	0.0194		III	0.007	0.0615		III	0.043	0.0446
	II	0.032	0.0355		I+II	(0.28,0.72)	0.0148		IV	0.065	0.0396
	I+II	(0.67,0.32)	0.0097		IV	0.085	0.0403		V	0.068	0.047
	III	0.057	0.0359		V	0.087	0.047	Au_{60}^-	I	0.000	0.0105
	IV	0.119	0.0556		VI	0.124	0.0502		II	0.012	0.0099
	V	0.127	0.0267						III	0.030	0.0120
	VI	0.254	0.0486						IV	0.031	0.0178
	VII	0.370	0.0543	Au_{42}^-	I	0.000	0.0203		V	0.110	0.0172
	VIII	0.486	0.051		II	0.148	0.0387		I+V	(0.62,0.38)	0.007
Au_{25}^-	I	0.000	0.0151		I+II	(0.64,0.36)	0.0133		VI	0.136	0.0171
	II	0.030	0.0302		III	0.288	0.0354		VII	0.178	0.0125
	I+II	(0.66,0.34)	0.0084		IV	0.302	0.0243		VIII	0.203	0.0263
	III	0.113	0.0291		V	0.424	0.0238	Au_{62}^-	I	0.000	0.0249
	IV	0.136	0.0318	Au_{47}^-	Ia (triplet)	0.000	0.0127		II	0.011	0.0159
	V	0.259	0.0396		Ib (singlet)	0.002	0.0178		III	0.025	0.0074
Au_{27}^-	I	0.000	0.0566		Ia+Ib	(0.66,0.34)	0.011		II+III	(0.29,0.71)	0.0056
	II	0.069	0.0391		II	0.432	0.0234		IV	0.074	0.0129
	I+II	(0.27,0.73)	0.0353		III	0.527	0.0237		V	0.133	0.0137
	III	0.158	0.0723		IV	0.683	0.0235		VI	0.330	0.0235
	IV	0.186	0.0648	Au_{48}^-	I	0.000	0.0061	Au_{63}^-	I	0.000	0.0063
	V	0.296	0.0767		II	0.171	0.0206		II	0.198	0.0167
Au_{29}^-	I	0.000	0.0231		III	0.244	0.0208		III	0.277	0.0232
	II	0.033	0.034		IV	0.261	0.0178	Au_{64}^-	I	0.000	0.0148
	I+II	(0.59,0.41)	0.0112	Au_{49}^-	I	0.000	0.0196		II	0.021	0.0281
	III	0.110	0.0344		II	0.218	0.0371		III	0.039	0.0243
	IV	0.217	0.036		III	0.243	0.0372		IV	0.051	0.0208
	V	0.231	0.0298		IV	0.264	0.0447		V	0.056	0.0262
Au_{32}^-	I	0.000	0.0195	Au_{51}^-	I	0.000	0.0066	Au_{67}^-	I	0.000	0.0214
	II	0.001	0.0173		II	0.081	0.0391		II	0.025	0.0323
	III	0.021	0.007		III	0.168	0.0451		III	0.039	0.0428
	II+III	(0.10,0.90)	0.0068		IV	0.204	0.0272		IV	0.056	0.0381
	IV	0.021	0.0247	Au_{52}^-	I	0.000	0.0052	Au_{69}^-	I	0.000	0.0364
	V	0.103	0.056		II	0.291	0.0112		II	0.022	0.0246
	VI	0.125	0.0197		III	0.346	0.0177		I+II	(0.17,0.83)	0.0240
	VII	0.204	0.0295		IV	0.383	0.0304		III	0.030	0.0319
Au_{33}^-	I	0.000	0.0077	Au_{55}^-	I	0.000	0.0085		IV	0.138	0.0543
	II	0.061	0.0216		II	0.080	0.0371		V	0.162	0.0398
	III	0.065	0.0288		III	0.142	0.034				
	IV	0.095	0.0156		IV	0.189	0.0205				

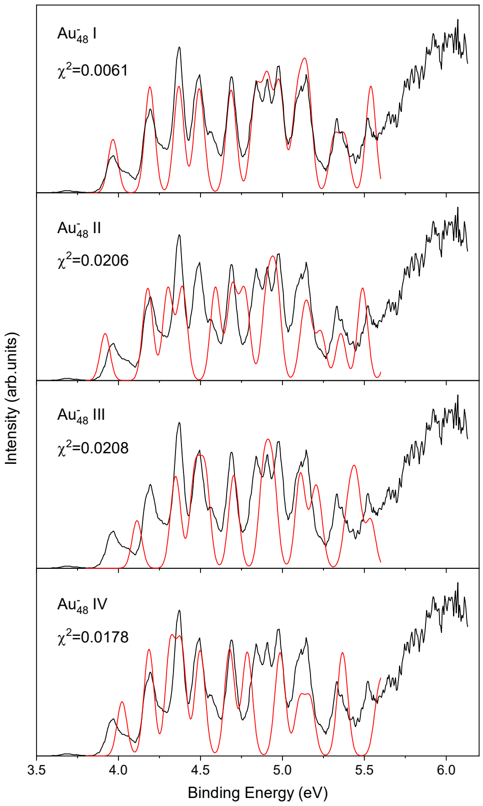

Supplementary Figure 7: The EDOS of low-energy isomers of Au_{48}^- (red lines) fitted to the experimental photoelectron spectrum (black lines). The quality of the fit is quantified by a χ^2 value.

Suppl. Note 5. Entropy induced hidden global minima

Our ability to accurately match the photoemission spectra, exclusively using the EDOS of the most stable structures located at the PBE0-SO-D3 level of theory, implies that theory provides reasonably accurate stabilities. This comment is particularly relevant in cases where the EDOS of an excited isomer is the one that best matches the PES, while the GM structure does not provide the best possible match. It is then important to complement the theoretical energies with configurational entropies in order to properly estimate the equilibrium population of different structures with similar energies in the experimental cluster beam. If the GM structure is highly symmetric (the order h of its point group is large), and if there are competitive isomers with lower symmetry, the configurational entropy contribution tends to decrease the percentage equilibrium population of the global minimum structure. If the effect is sufficiently large, the equilibrium population of the more symmetric structure might

become negligible, implying that the GM structure is "hidden" because of the entropic advantage of the lower symmetry structures.

An accurate theoretical estimation of equilibrium populations is hard to obtain, as they depend exponentially on the energy differences between isomers through the Boltzmann factor, as well as on the differences in the vibrational densities of states. Nevertheless, in this section we show some examples that demonstrate that the PBE0-SO-D3 thermochemistry has at least semi-quantitative accuracy for predicting equilibrium populations of isomers that agree with the experimental PES measurements. More in detail, we have estimated the equilibrium populations at room temperature for those sizes for which the GM structure seems to be hidden. In choosing room temperature as a representative temperature, we are assuming, as a working hypothesis, that structural isomerizations will not be fast enough below room temperature, i.e. there will be kinetic trapping of structures on the experimental time scale. This could be rationalized considering that the Debye temperature of gold (170 K) is quite high, meaning that room temperature in fact is a rather low temperature for gold. Of course, repeating the calculation for different temperatures will modify the predictions, which is another way of saying that our estimations have at most semi-quantitative accuracy.

As a representative example, we discuss the case of Au_{32}^-. The GM structure has D_{3h} symmetry, while the competitive isomers II, III and IV have, respectively, C_2, C_1 and C_s symmetries (isomer IV is not explicitly shown in Supplementary Fig. 5 even if it has a competitive energy, because its EDOS is not explicitly needed to match the PES). The point group orders are $h = 12$ for D_{3h}, $h = 2$ for C_2 or C_s, and $h = 1$ for C_1. This implies, for example, that the number of different permutation-inversion versions of the C_1 isomer (different local minima on the potential energy surface, including the enantiomers in the case of chiral symmetries) is 12 times larger than for the GM structure with D_{3h} symmetry. On a more dynamical picture, it implies that there are 12 different paths on the potential energy surface connecting the D_{3h} structure with the C_1 distortions, while there is only one path for the reverse isomerization transition. Using the calculated energies (and assuming similar vibrational frequencies for all isomers), the relative populations at room temperature are predicted to be 7%, 39%, 36% and 18% for isomers I to IV. Thus, one expects that the population of the highly symmetric GM is reduced by entropy: isomers II and III should be the dominant species in the experimental sample, present in approximately equal amounts, while both IV and the true GM should contribute comparatively less to the PES. Indeed Fig. 2 suggests that isomer I is not dominant, the spectrum can be fully explained by contributions from isomers II and III. Isomer III turns out to be more abundant than isomer II, which hints at a slightly higher energy of isomer II than the one calculated or at an additional influence of vibrational entropy.

Similarly, for Au_{39}^-, the twisted pyramidal isomers in Supplementary Fig. 5 have C_1 symmetry and, therefore, each of them is four times more abundant than the C_{2v} structure (isomer III) in terms of non-superimposable local minima in the potential energy surface. Given the very small energy differences, the estimated room-temperature equilibrium populations are 48% , 43% and 9%, for isomers I, II and III, respectively. As

the two families are now very different, it is even more sensible to assume that interconversion will not be easy below room-temperature - the isomer population might even freeze in at higher temperatures. In conclusion, the theoretical prediction is that twisted pyramids should be dominant in the experimental sample for Au_{39}^-, with the C_{2v} structure being present as a minority species only. Once more, this prediction agrees with the experimental PES (see Fig. 2 of the main text). All other sizes with entropy induced hidden minima can be discussed on similar grounds.

One should note that this discussion neglects possible differences in vibrational entropy, which could similarly influence the isomer population. If the vibrational density of states is significantly different for different isomers, the isomer with overall lower vibrational frequencies (leading to a larger heat capacity at low temperatures) will have a larger vibrational entropy around room temperature and therefore an enhanced equilibrium abundance. For isomers belonging to the same structural family one can assume rather similar vibrational frequencies; for very different families, however, this might not be the case. This is, therefore, an aspect which certainly warrants further work.

Suppl. Note 6. Additional discussion on the role of dispersion, non-local exchange, spin-orbit and other effects

The delicate balance between non-local exchange, dispersion, and spin-orbit effects is key to obtain an accurate thermochemistry, allowing the identification of the correct GM structures. The attractive effects of dispersion systematically stabilize structures with a denser atomic packing (i.e. a higher average coordination number), while non-local exchange effects (PBE0) do exactly the opposite. Accounting for both effects in a transferable and accurate way is therefore essential to obtain, for example, the correct number of core atoms in the GM structures. The spin-orbit contribution to cluster stability is also essential to determine the GM structure for some sizes, but its effect is much less systematic or predictable. Concerning spectroscopic properties, non-local exchange and spin-orbit effects are both crucial ingredients to make the EDOS meaningfully comparable to the photoemission spectrum. The D3 dispersion correction can also be considered essential for spectroscopic properties, even if its effect on the EDOS is of an "indirect" nature, i.e. by selecting the right GM structure whose EDOS should match the PES. In this section, we discuss some explicit examples of the outmost importance of the dispersion energy contribution.

For Au_{21}^- we observe a competition between bi-layer structures based on a hexagonal prism or antiprism unit (isomers I, II and III) and pyramids with adatoms (for example, isomer V in Supplementary Fig. 5), all of them containing no internal atoms. Pyramids are less compact (less densely packed) than bi-layers in terms of average atomic coordination number. As a result, the relative stability of these two families strongly depends on the level of theory employed. Without accounting for dispersion effects, PBE0-SO would assign a pyramid with a low coordinated adatom as the most stable structure, because non-local exchange stabilizes the less compact pyramids a lot. The EDOS of isomer V, shown in Fig. 2 of the main text, matches many of the strong

signals in the PES, but not all of them, so it can not be the correct GM structure. Dispersion effects clearly destabilize the pyramids with respect to bi-layers, and destabilize dangling atoms in general as well, so it is essential to reach consistency between experimental measurements and theoretical predictions for that particular size.

As shown in the main text, dispersion is even more important when comparing the stabilities of structures with a different number of core atoms, because it systematically stabilizes the isomer with a larger number of core atoms. Au_{25}^- is a representative example. PBE0-SO would assign structure II, a member of the bi-layer family without internal atoms, as the single dominant isomer, being 0.3 eV more stable than the true GM structure at that level of theory. PBE0-SO-D3, however, predicts that structures I and II are nearly-degenerate, with isomer II being 0.03 eV less stable than the GM structure. Here dispersion is essential to obtain an atom-filled structure as the GM. The transition towards structures with one core atom therefore occurs at $N = 25$, a prediction confirmed by the quality of the EDOS/PES match in Fig. 2 of the main text. In general, the dispersion energy contribution is essential to predict the critical sizes at which the number of core atoms increases. Similar situations occur, for instance, for Au_{33}^-, the smallest cluster with four core atoms; Au_{49}^-, the smallest size with 7 core atoms; or Au_{58}^-, the smallest anion with a full Ih_{13} icosahedral core. In all of these cases, the most stable structures obtained by neglecting the dispersion energy have a smaller number of core atoms, but can be excluded as the corresponding EDOS do not match the photoemission spectra. Particularly illuminating is the Au_{49}^- case, where the GM structure according to the PBE0-SO level of theory would still be a bi-pyramidal fragment with only five core atoms, similar to the GM structure of Au_{48}^-. Introduction of the dispersion correction completely changes the energies of the most relevant local minima on the potential energy surface, stabilizing the chiral tubes over the bi-pyramidal fragments. Again, only the latter ones provide a good EDOS/PES match. There are additional examples, all of which can be discussed in similar terms - this will be done in more detail in forthcoming publications.

Suppl. Note 6.1. Dispersion destabilizes hollow cages and tubes for gold clusters

In recent years, several theoretical reports have predicted the existence of stable hollow structures (with no internal atoms) for gold clusters, in the form of rounded cages or elongated tubes. As examples, Liu *et al.*(18) predict a highly symmetric (D_{6d}) and hollow tubular isomer for size $N = 26$, which is obtained from our GM structure for Au_{27}^- after removing the core atom; the hollow icosahedral cage at size $N = 32$ has been extensively discussed as a highly stable isomer in several reports(19; 20; 21; 22; 23; 24; 25); Liu and Hamilton(26) reported a stable chiral cage for Au_{42}^-; finally, Garzón and coworkers have studied a hollow chiral icosahedron for $N = 60$(27). We notice that none of these studies include an explicit account of the dispersion energy contribution. In this work, we have demonstrated that including the dispersion energy is essential to obtain the correct GM structure and the relative stabilities of structural

Supplementary Table 7: Total energy differences between the hollow isomer and the GM structure (ΔE), and dispersion contribution ($\Delta E(D3)$) to those total energy differences, are shown in eV units.

Cluster	ΔE (eV)	$\Delta E(D3)$ (eV)
Au_{26}^-	0.363	0.527
Au_{32}^-	1.485	1.539
Au_{42}^-	3.128	2.563
Au_{60}^-	16.261	8.192

isomers. It is therefore interesting to analyze the quantitative effect of the dispersion interactions on the stability of hollow cage structures.

We have fully optimized the hollow isomers of Au_{26}^-, Au_{32}^-, Au_{42}^- and Au_{60}^-, at the PBE0-D3 level of theory (i.e. not relying on the single-point PBEsol approximation). Supplementary Table 7 displays the relevant results: the energy difference ΔE between the hollow isomer and the GM structure at PBE0-D3 level of theory, and also the dispersion contribution $\Delta E(D3)$ to that energy difference. The results demonstrate that all those hollow isomers are significantly less stable than the correct (atom-filled) GM structure.

The first expected observation is that the hollow cages become systematically more unstable the larger the cluster size, ΔE evolving from 14 meV/atom for $N = 26$ to 271 meV/atom for $N = 60$, and furthermore, $\Delta E(D3)$ is seen to be a decisive contribution to that systematic trend. But even more interesting is that the dispersion contribution for $N = 26$ and $N = 32$ is bigger than the total energy difference, i.e. the hollow cages would be more stable at plain PBE0 level (in the absence of dispersion effects). This demonstrates that dispersion is essential for rendering the hollow cages with 26 and 32 atoms unstable; it also strongly contributes to the high energy of the two larger cages. We therefore expect our results to influence all future studies considering hollow cages: dispersion effects must be explicitly included.

Our results demonstrate that golden hollow cages are much more unstable on energetic terms than previously considered, if the significant dispersion contribution to the energy is properly accounted for. Even if the cages are mechanically stable (i.e. local minima on the potential energy surface with no imaginary normal mode frequencies), they will display a strong energetic bias towards densification into more compact structures with inner atoms. Therefore, we expect them to be quite difficult to synthesize and stabilize as isolated hollow cages, though they could perhaps be stabilized through interaction, for example, when encapsulating some endohedral dopants, in multi-shell structures, or if protected with external ligands.

Supplementary Table 8: Total energy differences (evaluated at PBE0-D3 level of theory and in eV units) between the sixteen more stable structures of Au_{55} identified in a previous EP/DFT computational work(28) and our GM structure. All the energies in this table refer to Au_{55}^- in its anionic form.

Isomer #	ΔE (eV)	Isomer #	ΔE (eV)
1	1.122	9	0.273
2	0.921	10	0.492
3	1.259	11	0.315
4	0.890	12	0.296
5	0.459	13	1.389
6	1.346	14	1.500
7	0.634	15	0.612
8	0.518	16	1.110

Suppl. Note 6.2. Importance of the parametrization of the empirical potential: Au_{55}^- as a test case

A previous computational work by Schebarchov and coworkers(28) contains a very detailed investigation of several neutral gold clusters, and of Au_{55} in particular, for which a disconnectivity graph with 500 isomers was generated. These authors employed an empirical potential (EP)/DFT strategy similar to ours, using a Gupta potential to extensively sample an approximate energy landscape and build the disconnectivity graphs, and then performing additional optimization of selected structures at the PBE-DFT level of theory. Although in strict terms neutral and anion clusters should not be directly compared, significant differences in the potential energy landscapes of Au_{55} and Au_{55}^- are not expected. Therefore, in this subsection, we compare our results for Au_{55}^- with their results for Au_{55}.

It is important to emphasize that Schebarchov *et al.* employed a Gupta potential for generating trial structures like we have done, but it is a Gupta potential with parameters significantly different from those in our optimized Gupta potential, so strong differences could be expected even at the EP level. It is the influence of the EP parametrization that we focus on in this subsection.

Schebarchov *et al.* re-optimized their 16 most stable Gupta structures at the DFT-PBE level, and found some energy reordering: the ninth isomer according to their Gupta energies becomes the most stable structure at the DFT-PBE level, which is significantly different from our GM structure. To analyze the origin of the discrepancy, we have evaluated the energies of these 16 isomers at PBE0-D3 level and for Au_{55}^- in its anionic form, in order to compare their energies with our putative GM energy for the anion. The results are provided in Supplementary Table 8, where ΔE is the energy difference with respect to our putative GM energy, and should be compared with the DFT-PBE values reported in Table 1 of their paper(28).

A first observation is that the energetic ordering of these 16 isomers is quite similar to what the authors predicted based on their PBE calculations. In particular, we also find that the ninth isomer in their Gupta list is the most stable one at the PBE0-D3 level out of those 16 structures, and even the relative energies of the different isomers are in quite fair agreement with the results by Schebarchov *at al.*(28); therefore, their use of PBE (instead of PBE0-D3) does not seem to be the main origin for the discrepancy, nor their consideration of neutral clusters instead of charged ones. The most important point is that even the most stable one of these isomers is almost 0.3 eV above our putative GM energy. In fact, their putative GM structure does not even belong to the structural family of frustrated icosahedra (though it does have 10 core atoms, as the correct GM has). We conclude that the main origin of discrepancy is the use of a substantially different Gupta potential to generate tentative initial structures. That is our explanation for the observation that all of their more stable Gupta structures are in a high energy region of the PBE0-D3 potential energy landscape (their Gupta potential energy surface differs too much from the more realistic PBE0-D3 potential energy surface, so even an extensive sampling of it does not provide competitive initial structures).

Suppl. Note 6.3. Comments on the performance of PBEsol functional

We close this section with a discussion of the rather good performance of the PBEsol functional observed in this work, which leads us to suggest it as the GGA of choice for a fast scanning study of gold clusters. As Fig. 1 of the main text demonstrates, PBEsol can not accurately describe the electronic density of states, irrespective of the inclusion of spin-orbit effects, so the PBE0-SO-D3 calculations are essential for structural assignment based on a EDOS/PES comparison. However, PBEsol produces very accurate geometries (see Suppl. Note 1.3), i.e. almost indistinguishable from the PBE0-D3 optimal geometries, and it also provides reasonable (i.e. useful in practice) relative stabilities of isomers: typical differences between PBEsol and PBE0-D3 relative stabilities of isomers are around 0.1 eV. Therefore, single-point PBE0-SO-D3 calculations, performed on the PBEsol optimal geometries and only for the more stable PBEsol structures suffice to obtain accurate stabilities of isomers and electronic properties such as the EDOS.

This very nice performance of PBEsol was unexpected and, in our opinion, is a consequence of a fortunate cancellation of errors. This functional was specifically designed(3) to improve lattice constants and surface energies of solids, but at the cost of worsening atomization energies. Being specifically optimized for solids, it is no surprise that PBEsol performs much worse than PBE for most molecules and clusters formed by metallic elements. In particular, atomization energies of many molecules are significantly overestimated by PBEsol(3). On the other hand, no GGA without explicit dispersion corrections can account for long-range correlation effects, which are very important for gold, as demonstrated in this work. We speculate that a particular error cancellation between exchange and correlation implemented in PBEsol can phenomenologically account for medium-range dispersion attraction, somehow mimicking (albeit in a non-transferable way) the effects of dispersion.

Suppl. Note 7. The number of core atoms in a spherical cluster

The radius of a close to spherical cluster with N atoms is

$$R = N^{1/3} r_{ws},$$

where r_{ws} is the Wigner-Seitz radius of the cluster material. Clusters with icosahedral, decahedral or octahedral symmetry show a dense atomic packing close to that of an fcc-lattice, with - except for truncations - (111)-like facets on the surface. The distance between (111) layers in a fcc-lattice is

$$d = \frac{1}{\sqrt{3}} a,$$

where a is the lattice constant. In a simple fcc-crystal with 4 atoms per unit cell one has

$$a^3 = 4 \frac{4\pi}{3} r_{ws}^3$$

or

$$a = \sqrt[3]{\frac{16\pi}{3}} r_{ws}.$$

In this case the distance between the (111) layers becomes

$$d = \frac{1}{\sqrt{3}} \sqrt[3]{\frac{16\pi}{3}} r_{ws}.$$

Assuming that this also is the distance between shells of atoms in a spherical cluster, the core atoms form a sphere with a radius of $R - d$. The number of core atoms in a spherical cluster therefore is

$$N_c = \left(\frac{R - d}{r_{ws}} \right)^3 = \left(N^{1/3} - 1.477 \right)^3.$$

This is the relation used for the dashed line in Fig. 4c). It is in fact close to the result for a cluster with icosahedral structure. Closed shell icosahedra with n shells have atom numbers of

$$N = -1 + \frac{11}{3} n - 5n^2 + \frac{10}{3} n^3.$$

The number of core atoms is thus

$$N_c = -1 + \frac{11}{3}(n-1) - 5(n-1)^2 + \frac{10}{3}(n-1)^3.$$

For large n this converges to

$$N_c = \left(N^{1/3} - \sqrt[3]{\frac{10}{3}} \right)^3 = \left(N^{1/3} - 1.494 \right)^3.$$

Supplementary references

[1] Aguado, A. *et al. Angew. Chem. Int. Ed.* **54**, 2111-2115 (2015).

[2] Aguado, A. *et al. Nanoscale* **10**, 19162-19181 (2018).

[3] Perdew, J. P. *et al. Phys. Rev. Lett.* **100**, 136406 (2008).

[4] Soler, J. M. *at al. J. Phys.: Condens. Matter* **14**, 2475 (2002).

[5] Troullier, N. & Martins, J. L. *Phys. Rev. B* **43**, 1993 (1993).

[6] Louie, S. G.; Froyen, S. & Cohen, M. L. *Phys. Rev. B* **26**, 1738 (1982).

[7] Kresse G. & Hafner, J. *Phys. Rev. B.* **47**, R558 (1993).

[8] Kresse, G. & Furthmüller, J. *Phys. Rev. B.* **54**, 11169 (1996).

[9] Makov, G. & Payne, M. C. *Phys. Rev. B.* **51**, 4014 (1995).

[10] Pašteka; Eliav, L. F. E.; Borschevsky, A.; Kaldor, U. & Schwerdtfeger, P. *Phys. Rev. Lett.* **118**, 023002 (2017).

[11] Baek, H. *et al. J. Phys. Chem. A* **121**, 2410-2419 (2017).

[12] Jackschath, C. *et al. Ber. Bunsenges. Phys. Chem.* **96**, 1200-1204 (1992).

[13] Takahashi, L. K. *et al. J. Phys. Chem. A* **113**, 4035-4044 (2009).

[14] Honeycutt, J. D. & Andersen, H. C. *J. Phys. Chem.* **91**, 4950-4961 (1987).

[15] Faken, D. & Jónsson, H. *Computer Materials Science*, **2**, 279–286 (1994).

[16] Larsen, P. M. *Revisiting the Common Neighbour Analysis and the Centrosymmetry Parameter*, arXiv:2003.08879 (2020).

[17] Aguado, A. & Kostko, O. *J. Chem. Phys.* **134**, 164304 (2011).

[18] Liu, Q. *at al. Nanoscale* **11**, 13227 (2019).

[19] Bulusu, S. *et al. PNAS* **103**, 8326-8330 (2004).

[20] Fa, W. and Gong, J. *J. Am. Chem. Soc.* **127**, 32-33 (2005).

[21] Johansson, M. P. *et al. Angew. Chem. Int. Ed.* **43**, 2678-2681 (2004).

[22] Häkkinen, H. *et al. Angew. Chem. Int. Ed.* **44**, 5244-5248 (2005).

[23] Bulusu, S. *et al. J. Phys. Chem. B* **109**, 15638-15642 (2005).

[24] Rauhalahti, M. *et al. RSC Advances* **6**, 17094 (2016).

[25] Zhao, W. *et al. J. Phys.: Condens. Matter* **34**, 224005 (2022).

[26] Liu, X. J. and Hamilton, I. P. *Nanoscale* **9**, 10321-10326 (2017).

[27] Mullins, S.-M. *et al. Nature Communications* **9**, 3352 (2018).

[28] Schebarchov, D. *et al. Nanoscale* **10**, 2004-2016 (2018).
