# Supplementary material for: Structural motifs of gold cluster anions with 17 to 69 atoms
Source: Nat Commun. 2026 Apr 10;17:5099. doi: 10.1038/s41467-026-71649-9 (PMC13246909; doi:10.1038/s41467-026-71649-9)
Supplement: Supplementary file 2 — Description of Additional Supplementary Information [file 41467_2026_71649_MOESM2_ESM.pdf]

Description of Additional Supplementary Files

File Name: Supplementary Data 1
Description: Coordinate files for all cluster structures presented plus DFT program input files.
