# Supplementary material for: Structural motifs of gold cluster anions with 17 to 69 atoms
Source: Nat Commun. 2026 Apr 10;17:5099. doi: 10.1038/s41467-026-71649-9 (PMC13246909; doi:10.1038/s41467-026-71649-9)
Supplement: Supplementary file 4 — Transparent Peer Review file [file 41467_2026_71649_MOESM4_ESM.pdf]

Peer Review File

Structural motifs of Gold Cluster Anions with 17 to 69 atoms

Corresponding Author: Professor Bernd von Issendorff

This file contains all reviewer reports in order by version, followed by all author rebuttals in order by version.

Version 1:

Reviewer comments:

Reviewer #1

(Remarks to the Author)
The authors accurately obtains the globally most stable structure of medium-sized Au17-69 clusters by considering spin-orbit and dispersion effects in the functional. The theoretical simulated photoelectron spectra align well with the experimental photoelectron spectra. What is particularly remarkable is that, across a wide size range from 17 to 69 atoms, the theoretical spectra consistently match the experimental ones.
This is a valuable reference in current cluster research, where typically only one or two theoretical methods are used to study one or two clusters, and theoretical results are assumed to precisely describe the cluster behavior. A functional theory that can well describe cluster systems must be applicable to the majority of cluster sizes. Therefore, this is an excellent piece of work.I recommend that this article be accepted.

The reviewer still has some questions:
1. The literature suggests that larger Au clusters are prone to forming amorphous structures, but this seems to be cited in only one paper and is not a mainstream viewpoint. Are there more studies supporting this?
2.When searching for the configurations, did the author consider the effect of temperature on the configurations? In experiments, Au clusters are cooled by a molecular beam, and the actual isomer distribution follows a Boltzmann distribution. Did the author account for this in their theoretical calculations?
3.3. Is the author's theoretical model applicable to doped clusters? For example, to transition metal-doped Au cluster systems.

Reviewer #2

(Remarks to the Author)
The manuscript by Aguado, Issendorf and coworkers is a valid example of a combination of experimental data to define a robust theoretical/numerical procedure to obtain detailed information on the structure of gold clusters within the subnanometer range. I suggest major revisions

The manuscript has the potential to define a strategy although the importance of relativistic effects for Au clusters are not new to the community.
Related to that I see a lack of comparison against available data regarding cage and tubular shape predicted for Au clusters at 26,32,42,52 atoms (neutral). See for example, Liu et Al. Nanoscale(2019) a tubular D6d for 26 atoms. The work by Liu et Al. On the stability of chiral tubular for Au with 9n+6 atoms and n>1 (Liu and Hamilton nanoscale (2017))
Especially the golden fullerene at 32 atoms have shown a significant stability for both neutral and charged cases (see Johannson Agee. Chen. (2004), Baletto et Al PCCP (2015), Zhao et Al. J.Phys.-cond.Matt.(2022)). But search for the 42 and 52 atoms too. See work by Mironov and Alvaro Munoz-Castro. A comparison with isomers for Au55 by Wales and coworkers is highly appreciated (Schebarchov Nanoscale (2018)).
Without the inclusion of cage like isomers the work can not be published as the search is biased by the use of the Gupta potential as first screening.
In addition, the authors should clarify the following points
* A key point is whether there is any expectation that Au clusters formed in a liquid nitrogen magnet on sputtering gas aggregation source to be in their lowest energy isomer.
In any event I would appreciate a deeper explanation why not always the lowest isomer yields the EDOS/PES. Including experimental justifications.
* In supplementary information the authors must add a more complete comparison between SIESTA and VaSP. What is the

rationale of using a localised and a plane wave code?
* The authors must provide in a database the identified cluster shape. More geometrical description is needed for example looking at the pair distance distribution function, common neighbour analysis etc.
* I will suggest to improve the graphics of the figures to make them more attractive: one to show the strategy and the other to have isomers and PES.
* the authors should comment on which materials is possible to compare PES and EDOS.
* the shift of about 0.3 eV must be explained better especially for a claim that PBE0 with SO-D3 is the right scheme to use.
* add a Suppl Info note on experimental data and whether they do refer to individual nanoparticle or to a sample.

Reviewer #3

(Remarks to the Author)
A large amount of work has been done over the last two decades to identify the structures of gold clusters in the gas phase. While the structures of small gold cluster anions (up to ~Au_{20}^-) are well understood, a consistent picture of the ground state structures of medium-size clusters, as evidenced by agreement with experimental data, is still lacking for many cluster sizes.

The work of Aguado et al. provides (re)assignment of gold cluster anions over a wide size range (17–69 atoms) by comparing the electronic density of states (EDOS) obtained from density-functional calculations with photoelectron spectra.

The methods employed in this study are similar to those used in previous studies (Refs. 34, 35 and 36). The main differences are the use of different functionals for structure optimization (PBEsol) and for computing the density of states (SO-PBE0-D3). The latter includes an explicit dispersion term that favors higher-symmetry structures, many of which have not been identified before.

The choice of the DFT variants is well justified and documented, the structure assignment by comparison of EDOS and PES is convincing.

The paper is of excellent scientific quality and certainly deserves publication. However, I think this work is too specialized for Nature Communications. I suggest publishing it in a more focused journal, such as Nature communications chemistry or Nanoscale, provided that the authors address the following suggestions and comments, which are mostly minor.

1. Line 110 ff and fig. 1: What are the EDOS shifts used here? Pls. provide EDOS shifts for each functional in panels.
2. Line 133“In most cases the calculated EDOS of the ... ground state structure is in excellent agreement with the ... photoelectron spectra.” Fig 2 as well as paragraph 1.3 and Table4 SI present a different picture: in many cases more than one isomer is necessary to describe the PES. Pls. comment.
3. Line 269 : “perfectly iscosahedral” together with “local rearrangements” exclude each other.
4. Line 370: Au57- was identified before as a “bubble” structure, now its termed “well packed”. This seems not consistent, pls. comment.
5. Line 469: “PBEsol... (is) providing very accurate cluster geometries”: Please give a reference/refer to the discussion in SI.
6. Fig.2 is the basis of the structure assignment. However, it’s not clear to me how exactly the EDOS shown is generated. In detail: pls. explain how the EDOS of the different isomers are scaled.
7. Fig. 3: Alternative views of the clusters are provided for certain cluster sizes. Please indicate the relative orientation in the figure. When different isomers are shown, it is helpful to provide their relative energy.
8. Fig3: Some higher-lying isomers are necessary in order to explain the PES (Au25 II, Au32 II, III, and others). These are only shown in the SI. However, the reader should be able to follow the paper without constantly looking at the SI, so I suggest showing all the relevant structures in Fig.3
9. P18 SI: The GM for Au39- seems significantly different from the structural motif of the PES fitting isomers II and III. This makes the "configurational entropy only" assumption questionable. Pls. comment.
10. SI Table 4: When two isomers are required to fit the experimental data, what are their respective contributions?

Reviewer #4

(Remarks to the Author)
The manuscript presents an extensive DFT-based study on gold cluster anions in the size range N=17-69, identifying ground-state structures and comparing computed EDOS with experimental PES with an excellent match. The authors emphasize the role of relativistic effects, dispersion, and exchange interactions in achieving accurate predictions. The work is substantial and backed by robust benchmarking. However, some minor concerns should be addressed to improve clarity and strengthen the conclusions:

1. For those clusters where the best agreement with the experimental PES arises from a low-energy isomer rather than the calculated ground state, such as Au27-, Au32-, and Au39-, have the authors verified that structural reordering does not occur when full relaxations are performed at the PBE0-SO-D3 level? None of these structures are included in Table S1, which shows the accuracy of the SP energy calculations on PBEsol-optimized geometries. Given the likely flatness of the potential energy surfaces in these cases, it would be important to confirm that the use of different levels of theory for SP energies and geometry optimization does not lead to inconsistencies in energetic ranking or structural assignments.

2. The authors suggest that their method is transferable to ligand-stabilized gold nanoclusters. Have the authors performed any validation with such systems? Could they comment on how ligands (for example, thiolates) might alter the structural

trends they report? Even a single comparison with one of the most studied systems, such as Au25(SR)18- could support the claim of transferability.

3. Regarding the figures:

a) Figures showing the comparison of EDOS vs PES could be improved by adding a quantitative error measure. Adding vertical lines for key peak positions would also aid the reader.
b) Labels for symmetry groups in Fig.3 are hard to read, and the color scheme for atom distinction is inconsistently described. The figure caption should be improved, and clearer labeling (with a higher font size) for the structures would be better.

4. Consider uploading xyz coordinates as part of the SI material.

In general, the manuscript presents a robust methodology, is well-written, and is supported by a detailed analysis for a wide range of structures. I believe this study is a strong contribution to the field and would be suitable for publication once the authors address the concerns outlined above.

Version 2:

Reviewer comments:

Reviewer #1

(Remarks to the Author)
There are no further issues with the response to the review comments or the revised manuscript. The necessary revisions have been adequately addressed, and I am satisfied with the changes. Therefore, I agree to accept it for publication.

Reviewer #2

(Remarks to the Author)
I appreciate the revision work done by the authors. The bibliography in the main text should include the references suggested.

Probably the manuscript will befit better the Nature style if the authors add a pictorial representation of the numerical workflow.
Minor English typos and syntax that can be done during the proof reading

Reviewer #3

(Remarks to the Author)
In the revised version the authors have answered my questions and addressed my concerns, and I believe the same applies to the other reviews. I support publication in Nature Communications; the article can be published basically in its current form. Only a few minor points remain:

1. Pls. update the data availability statement with regard to the coordinate files.
2. Page 22 SI: ‘isomers II and III ... present in approximately equal amounts...’ is inconsistent with SI. Tab. 6 : ‘Au32-: II+III (0.1, 0.9)’
3. SI: pls. check citation of reference [13]

Reviewer #4

(Remarks to the Author)
The authors have adequately addressed most of my concerns by including new data with a very detailed discussion. Also, new adjustments in the text and figures clarify their interpretation of the results. Thus, I recommend the manuscript for publication in its current form.

Open Access This Peer Review File is licensed under a Creative Commons Attribution 4.0 International License, which permits use, sharing, adaptation, distribution and reproduction in any medium or format, as long as you give appropriate credit to the original author(s) and the source, provide a link to the Creative Commons license, and indicate if changes were made.
In cases where reviewers are anonymous, credit should be given to 'Anonymous Referee' and the source.
The images or other third party material in this Peer Review File are included in the article's Creative Commons license, unless indicated otherwise in a credit line to the material. If material is not included in the article's Creative Commons license and your intended use is not permitted by statutory regulation or exceeds the permitted use, you will need to obtain permission directly from the copyright holder.
To view a copy of this license, visit https://creativecommons.org/licenses/by/4.0/

We thank the referees for their detailed and helpful comments and questions. In the following we respond to all of the questions and describe the results of our additional calculations as well as the changes we made to the manuscript and the SI.

Reviewer #1

1. The literature suggests that larger Au clusters are prone to forming amorphous structures, but this seems to be cited in only one paper and is not a mainstream viewpoint. Are there more studies supporting this?

The referee is correct, one could indeed argue that this is no longer a mainstream viewpoint, but it certainly was so in the past, and it is still present in rather recent studies, like for example in

N. Tarrat, M. Rapacioli, and F. Spiegelman, J. Chem. Phys. **148**, 204308 (2018).

K. Sukuta, M. V. den Bossche, A. Pedersen, and H. Jónsson, Physics, Chemistry, Mathematics **8**, 723 (2017).

With our statement we just wanted to make clear that this former mainstream viewpoint is not valid anymore – notwithstanding the fact that this is hardly a surprise for many experts.

In any case, we have added two more references stating a tendency of gold clusters towards disorder to the main text (refs. 31 and 32).

2. When searching for the configurations, did the author consider the effect of temperature on the configurations? In experiments, Au clusters are cooled by a molecular beam, and the actual isomer distribution follows a Boltzmann distribution. Did the author account for this in their theoretical calculations?

This is a very interesting question, and in fact not easy to answer. In the gas aggregation process the clusters grow by attaching atoms; each attachment inserts the binding energy of roughly 3.5 eV into the cluster, heating, e.g., a size 40 cluster by 340 K. The clusters are constantly cooled by collisions with the buffer gas atoms (helium and argon at a pressure of about 0.5 mbar and a temperature of about 120 K), but only on a microsecond time scale, meaning that after each atom attachment the clusters have ample time to explore configuration space. With decreasing temperature isomerization will get slower and slower; at some temperature it will stop completely, which then freezes the isomer distribution present at that temperature. We therefore estimated the isomer distribution (based on energy differences and configurational entropy) by assuming the freeze-in temperature to be room temperature – it might in fact be quite a bit higher. This depends on the barrier heights between different structures, which are difficult to guess. More precisely, the barrier height will depend on the amount of reorganization necessary to change from one structure to another, which means that isomerization between very different structural families will have higher barriers and therefore stop at significantly higher temperatures than isomerization within a structural family. In such cases of competitive but rather different structural families the isomer population would even freeze into a distribution quite far from any thermal one.

We have shown in the SI that when assuming population freezing to occur at room temperature some high symmetry ground state structures can become “hidden” – this will be even more the case for higher freeze-in temperatures.

Measuring isomerization rates between different isomers as a function of temperature could be an optimal tool for determining relative energies of isomers and the relevant barrier heights; this will be tried in future experimental studies.

3. Is the author's theoretical model applicable to doped clusters? For example, to transition metal-doped Au cluster systems.

This is a very relevant question, but hard to answer in advance. As long as PBE0-D3 continues to provide a good description of the involved transition metal atoms, which probably is the case for most of them, it can be expected to also describe the doped gold cluster well, but this will have to be checked in each case.

Reviewer #2

1. Related to that I see a lack of comparison against available data regarding cage and tubular shape predicted for Au clusters at 26,32,42,52 atoms (neutral). See for example, Liu et Al. Nanoscale(2019) a tubular D6d for 26 atoms. The work by Liu et al. on the stability of chiral tubular for Au with 9n+6 atoms and n>1 (Liu and Hamilton nanoscale (2017)). Especially the golden fullerene at 32 atoms have shown a significant stability for both neutral and charged cases (see Johannson Agee. Chen. (2004), Baletto et Al PCCP (2015), Zhao et Al. J.Phys.-cond.Matt.(2022)). But search for the 42 and 52 atoms too. See work by Mironov and Alvaro Munoz-Castro. ….. Without the inclusion of cage like isomers the work can not be published as the search is biased by the use of the Gupta potential as first screening.

We thank the referee for his/her comment, because it motivated us to provide some additional examples of the importance of the dispersion contribution to cluster stability. Our paper clearly demonstrates that dispersion effects are essential to obtain a consistent description of gold properties from the dimer to the bulk limit (Table 1 in the report) and also to locate the correct GM structure that is able to match the experimental photoemission data. And it just happens that the dispersion effects render all the hollow cages mentioned by the referee uncompetitive for gold clusters. It is true that the Gupta potential does not locate hollow structures for the sizes mentioned, but, as stated in the report, we do not only test candidate structures obtained from the Gupta search, but also other structures taken from the literature or constructed by hand.

In addition to former structural screening mainly done at the PBEsol level, we have now fully optimized the hollow isomers of Au26- Au32- and Au42-, as mentioned by the referee, at the PBE0-D3 level. We have additionally included the hollow chiral icosahedron for Au60- predicted in a recent study by Garzon and coworkers [Nat. Comm. 9, 3352 (2018)]. The results are described in the new Subsection 6.1 in the Supplementary Information and summarized in the new Supplementary Table 7 (shown below). In this table we list the energy differences between the hollow cages and the GM structures at the PBE0-D3 level of theory, as well as the dispersion contribution to the energy differences:

	Delta_E (eV)	Delta_E (D3) (eV)
Au26-	0.363	0.527
Au32-	1.485	1.539
Au42-	3.128	2.563
Au60-	16.261	8.192

This demonstrates that dispersion is essential for rendering the hollow cages with 26 and 32 atoms

unstable; it also strongly contributes to the high energy of the two larger cages. We therefore expect our results to influence all future studies considering hollow cages: dispersion effects must be explicitly included.

2. A comparison with isomers for Au_{55} by Wales and coworkers is highly appreciated (Schebarchov Nanoscale (2018)).

The work mentioned by the referee contains a very detailed investigation of several gold clusters, and of Au_{55} in particular, for which a disconnectivity graph with 500 isomers was generated. It is important to mention that neutral Au_{55} was considered in that work, so the structures found may well differ from those of cluster anions. It is also important to mention that the authors of that work employ a Gupta potential for generating trial structures like we have done, but it is a Gupta potential with parameters significantly different from those in our optimized Gupta potential, so strong differences could be expected.

The authors re-optimized the 16 most stable Gupta structures at the DFT-PBE level, and found some energy-reordering: the ninth isomer according to the Gupta energies becomes the most stable structure at the DFT level. We have evaluated the energies of those 16 isomers at the PBE0-D3 level and for Au_{55} in its anionic form, in order to compare their energies with our putative GM energy for the anion. The results are provided in the new SI in tabular form (Supplementary Table 8 in the new Subsection 6.2) ; DeltaE is the energy difference with respect to our putative GM energy.

Gupta isomer	DeltaE(eV)
1	1.122
2	0.921
3	1.259
4	0.890
5	0.459
6	1.346
7	0.634
8	0.518
9	0.273
10	0.492
11	0.315
12	0.296
13	1.389
14	1.500
15	0.612
16	1.110

A first observation is that the energetic ordering of these 16 isomers is quite similar to what the authors predict based on their PBE calculations. In particular, we also find that isomer 9 in the Gupta list is the most stable one, so their use of PBE (instead of PBE0-D3) does not seem to be the origin of the discrepancy, nor the consideration of neutral clusters instead of charged ones. The important point is that even the most stable one of these isomers is almost 0.3 eV above our putative GM energy. In fact, the structure of "isomer 9" does not even belong to the structural family of frustrated icosahedra

(though it does have 10 core atoms, as the correct GM has). We believe that the main origin of discrepancy is the use of a substantially different Gupta potential to generate tentative initial structures. This is our explanation for the observation that all of the more stable Gupta structures are in a high energy region of the PBE0-D3 potential energy landscape, meaning that their Gupta potential energy surface differs too much from the more realistic PBE0-D3 potential energy surface, so even an extensive sampling of it does not provide competitive initial structures.

3. A key point is whether there is any expectation that Au clusters formed in a liquid nitrogen magnetron sputtering gas aggregation source to be in their lowest energy isomer. In any event I would appreciate a deeper explanation why not always the lowest isomer yields the EDOS/PES. Including experimental justifications.

As mentioned above, during formation the clusters get heated by atom attachment to appreciable temperatures and only slowly cooled by collision with the cold buffer gas. While at temperatures close to the melting point rapid isomerization can be assumed, at lower temperatures the isomer distribution will "freeze in", thus reflecting an equilibrium distribution at that temperature. At finite temperatures the configurational entropy can significantly influence the isomer distribution; as explained in the SI, high symmetry isomers can be suppressed despite being the lowest energy structures. The amount of suppression depends on the energy and entropy difference between the high symmetry ground state and that of higher lying (lower symmetry) structures, and on the barrier height between these structures, as this determines the "freeze-in" temperature. A somewhat similar influence of entropy has for example been described in Koskinen et al., PRL 98, 015701 (2007).

A direct experimental justification is not available currently. But we would like to stress that in cases where there is a clear ground state structure, we obtain a very good agreement of the EDOS of that structure with the experimental PES. Only in the cases where the ground state is highly symmetric and there exist low lying low symmetry structures, the agreement between the PES and the EDOS of the higher lying states is better – which means that the ground state structure has a low abundance in the experiment, as in fact can be expected based on the arguments given above.

4. In supplementary information the authors must add a more complete comparison between SIESTA and VaSP. What is the rationale of using a localised and a plane wave code?

The motivation for doing the PBEsol structural optimization of a substantial number of isomers with the SIESTA code was purely pragmatic: SIESTA is significantly faster than VASP for cluster systems (for a given required accuracy of energies and forces) so one can afford to do the required (many) PBEsol optimizations in a more reasonable time window. A localised basis code is in fact expected to be faster for simulations of finite cluster systems under a supercell approach, because a substantially smaller number of basis functions is required (as compared to the number of plane waves) to obtain a fixed given accuracy. At the starting time of this project, hybrid functionals were not available in the version of SIESTA with which we were working, and the treatment of spin-orbit effects, while included, was included only in an approximate way (under the so-called on-site approximation, which has limited accuracy in materials with strong hybridization such as gold). Otherwise, we would have opted for the highly efficient SIESTA code in all parts of the work. To summarize, we used our version of SIESTA as much as we could in this work for efficiency reasons, and turned to VASP only because of the necessity of using hybrid functionals and complete spin-orbit interactions. VASP also allowed us to do extensive functional testing (SIESTA does not include as many XC options as VASP).

Concerning the comparison between SIESTA and VASP, the higher computational efficiency of SIESTA comes at the cost of requiring careful basis set optimization to achieve the same level of accuracy. That the same level of accuracy can be achieved is demonstrated in several papers from the SIESTA developers (J. Phys.: Condens. Matter 14, 2745-2779 (2002), for example). We have ample experience in optimizing this type of basis sets and, as briefly mentioned in the Supporting Information, we carefully checked SIESTA/VASP consistency by performing explicit PBEsol calculations with the VASP code for a few test sizes.

In the new version of the SI document (Subsection 1.2), we have included a more quantitative comparison as requested by the referee. Specifically, we performed VASP optimizations at the PBEsol level for the global minimum structure of clusters with 21-26 atoms. For the optimal PBEsol/SIESTA geometry (SIESTA forces below 0.01 eV/Ang), the PBEsol/VASP forces were always below 0.03 eV/Ang. Upon PBEsol/VASP reoptimization, interatomic distances were modified by less than 0.01 Ang. These tiny modifications have negligible effect on the eigenvalue spectrum, the quantity of interest in our study.

5. The authors must provide in a database the identified cluster shape. More geometrical description is needed for example looking at the pair distance distribution function, common neighbour analysis etc.

We have now included the atomic coordinates of the global minimum structures, and also of the competitive isomers that contribute to the photoemission spectra, as part of the Supporting Information.

Concerning the additional geometrical description, we have also included the results of a common neighbor analysis of selected clusters from the frustrated icosahedral family in the SI (in the new Subsection 3.1). We agree that the distribution of pair distances contains very detailed information, which for example can be directly compared to the results of diffraction experiments. It is, however, relatively difficult to interpret in cases of mixed structural motifs. Therefore, we have opted for a common neighbor analysis (CNA), which may help to quantify the degree of icosahedral ordering present in what we call "frustrated icosahedral" structures.

As explained in the main text, we identified frustrated icosahedra by a detailed visual inspection of the structures, i.e. by assessing that the core of the cluster is an icosahedral fragment, and that the shell contains a significant amount of the pentagonal 16-atom caps that characterize the surface facets of a Mackay icosahedron in the size range around 55 atoms. We also included defects that are typically seen during icosahedral growth, such as the rosette defects, as part of our definition of "frustrated icosahedral" family. These purely visual descriptors clearly underline the structural trends that we wanted to emphasize.

We have now performed a CNA analysis for 5 sizes between n=55 and n=69 (the "frustrated icosahedra” size range), which clearly corroborates the partially icosahedral structure of these clusters.

These results are described in detail in the new subsection 3.1 of the revised Supporting Information.

6. I will suggest to improve the graphics of the figures to make them more attractive: one to show the strategy and the other to have isomers and PES.

If we understand the referee correctly, he/she suggests to add another figure explaining the structure search strategy; while this is certainly a good idea, we already reached the length limit of the article, so unfortunately there is no space available for an additional figure.

7. the authors should comment on which materials is possible to compare PES and EDOS.

Comparing PES and EDOS (of cluster anions) is one of the standard methods of cluster physics, and has been applied to a broad range of materials, like Na, Al, Mg, Zn, Cu, Ag, Au, Ti, V, Nb, Pd, C, Si (pure and doped), B, and others. Probably the best agreement up to date was achieved for Na, Al and Zn (refs. 43, 45, 47), but the agreement was never as good as the one now achieved for gold clusters. It is an interesting question whether the agreement can also be improved for other systems; one lesson learned from the gold clusters is that not only the geometrical structure has to be correct, but also the bond lengths have to be very precise in order to obtain a good EDOS/PES match - in the case of gold this could only be achieved taking dispersion into account, which might well be important for other materials as well. We hope that these findings will initiate similar efforts for other cluster systems.

8. the shift of about 0.3 eV must be explained better especially for a claim that PBE0 with SO-D3 is the right scheme to use.

We fully agree with the referee that an ideal calculation (using, in the case of DFT, the exact energy functional) should perfectly reproduce the electron affinity of gold clusters, and also of the gold atom, without the need for postprocessing shifts of any sort. The search for that "holy grail" functional is at the heart of the history of DFT, and we are certain that better functionals than the ones existing will emerge at some point. Searching for a functional that corrects the error in the electron affinity, without spoiling at the same time the good agreement that we find concerning all other properties tested in Table 1 and figures 1 and 2, is certainly an interesting avenue for new research, but it is beyond the scope of the present work. When we claim that "PBE0 with SO-D3 is the right scheme to use", what we mean is that it is presently the best option of the many ones that we explicitly tried. The identification and assessment of this level of theory will hopefully not be the last word on the subject, but is in our opinion a significant step forward for the computational research in gold clusters. There is already a paragraph (lines 510-525 in the original submission) explaining this issue, with a reference to the work by Pasteka and coworkers (reference 54 in the original submission), which contains a detailed explanation of what should be done in order to accurately calculate the electron affinity of the gold atom. Our work additionally demonstrates that the 0.3 eV shift observed in the gold atom is systematically transferred to all the gold cluster anions. There is little that we can presently add to that explanation, because a deeper analysis would imply starting a new and long-term investigation. Nevertheless, in the following, we try to elaborate a little bit more our arguments.

A first technical point to be mentioned is that we explicitly checked that the error in the electron affinity is not a consequence of dealing with a charged system under a periodic supercell approach, i.e. the error is not due to the technicalities of avoiding spurious electrostatic interactions between periodic replicas. In detail, we performed additional test calculations on the atom and small clusters up to the tetramer using the Turbomole code, which does not employ a supercell approach. We performed both DFT and CCSD(T) calculations, and found perfectly consistent results with VASP calculations at the same level of theory (DFT) or with the previous CCSD(T) results mentioned in Table

1 of our paper. We do not mention these tests explicitly in the paper because they are not central to the message of the work. We also emphasized in the paper that our DFT results are in very good agreement with CCSD(T) calculations of the electron affinity, i.e. even highly accurate calculations such as CCSD(T) are wrong by around 0.3 eV for that specific quantity of the gold anion. In fact, our detailed benchmarks show that PBE0-SO-D3 shows competitive performance when compared to CCSD(T) results: both PBE0-SO-D3 and CCSD(T) accurately predict all of the properties tested except the electron affinity value, where both levels of theory fail. Interestingly, the ionization potential of the neutral gold atom is accurately predicted in PBE0-SO-D3 and CCSD(T) calculations.

The work by Pasteka et al. (Ref. 10) has demonstrated how to reach meV accuracy for the IP and EA values of the gold atom. This requires not only a full relativistic treatment, but also taking all-electron correlation and even QED effects into account. For clusters such a treatment is currently not feasible. Nevertheless, our DFT calculations obviously do yield very good results for the bonding properties and the electronic structure of the clusters; only the electron affinity is systematically underestimated. This might be due to the incorrectly predicted asymptotic decay of the electron density at large distances. Global hybrids like PBE0 are known not to be able to recover the correct -1/r asymptotics of the XC potential, so we are quite confident this is the main source of the error. There are methods based on the XC potential (as opposed to the XC energy) that can be used to enforce the correct asymptotic decay of the electron density tail (works by Gritsenko and Baerends, for example J. Chem. Phys. 121, 655-660 (2004)). A real-time TD-DFT simulation of the photoemission process, in particular an analysis of the outward probability flux associated with the detaching electron, might also be useful for a better understanding of what occurs at large distances. This is a very interesting question and should be studied in more detail in the future. We can only emphasize once more that the focus of our work was on obtaining the best possible match between the EDOS and PES, and this has been achieved with PBE0-SO-D3, irrespective of the global shift, which we for now consider of secondary importance. Different functionals require different shifts (see our answer to question 1 of referee #3), but PBE0-SO-D3 proved to provide the best EDOS/PES match. In the new version of the SI, we have added some more details along these lines (the last paragraphs in Subsection 1.4).

9. add a Suppl Info note on experimental data and whether they do refer to individual nanoparticle or to a sample.

The experiments have been done on size-selected clusters in the gas phase, averaging photoelectron spectra over typically 30000 laser shots. The total number of clusters which have emitted a (detected) photoelectron typically lies between 10000 and 100000. In order to clarify this, we have added the following sentence to the methods section:

"Spectra were averaged over typically 30000 shots, and constitute averages of the kinetic energy distributions of photoelectrons detached from roughly 10000-100000 clusters."

Reviewer #3

1. Line 110 ff and fig. 1: What are the EDOS shifts used here? Pls. provide EDOS shifts for each functional in panels.

We have calculated the vertical detachment energy (VDE) of Au19- for all the functionals presented in Supplementary Figures 2 and 3, and have now indicated the corresponding applied global shift for each of them. The results demonstrate that all the hybrid functionals require similar global shifts to match the experimental photoemission spectrum. Even if some range-separated functionals such as HSE06 or HSEsol reduce the global error in the electron

affinity of Au19- as compared to PBE0, they worsen the agreement with experiment regarding the detailed structure of the PES. It is also interesting to notice that PBE0 and SCAN0, both having the same percentage amount of non-local exchange (25%), require roughly the same shift in the EDOS.

As stated in the previous version of the paper, none of the semilocal functionals is able to reproduce the photoemission spectrum. Nevertheless, it is interesting to notice that the SCAN meta-GGA approximation is the functional that best matches the experimental VDE value (Supplementary figure 2).

The additional data are briefly discussed in Section 2 of the revised Supporting Information.

2. Line 133"In most cases the calculated EDOS of the ... ground state structure is in excellent agreement with the ... photoelectron spectra." Fig 2 as well as paragraph 1.3 and Table4 SI present a different picture: in many cases more than one isomer is necessary to describe the PES. Pls. comment.

This is indeed correct – we changed the sentence to

"In most cases the calculated EDOS of the putative ground state structure (or a combination of those of the lowest and the next higher isomer) is in excellent agreement…"

We also changed line 76 to

".., in most cases exclusively employing the EDOS of the most stable structure (or of the two most stable ones) identified by theory.."

3. Line 269 : "perfectly icosahedral" together with "local rearrangements" exclude each other.

This is also a good point. What we were trying to state is that the strain inherent to icosahedral packing is released mainly by a local rearrangement of atoms, which for example leads to the formation of a flat facet on one side of the cluster. But as this releases the strain of the icosahedral part by allowing uniform bond lengths, indeed also the icosahedral part differs slightly from a perfect icosahedral structure. We therefore changed the sentences to

"where the structures are dominantly close to perfectly icosahedral, but release the stress through local atomic rearrangements in a small region of the cluster."

".. based on a 55-atom Mackay icosahedron, which is flattened on one side, but nearly perfectly icosahedral on the other side."

4. Line 370: Au57- was identified before as a "bubble" structure, now its termed "well packed". This seems not consistent, pls. comment.

Again correct. Indeed "packed" could be misunderstood as hinting at high density packing. We therefore replaced "packed" by "ordered":

"..it does not only have a closed electronic shell (fully filled 1G), but also a well ordered atomic structure"

5. Line 469: "PBEsol... (is) providing very accurate cluster geometries": Please give a reference/refer to the discussion in SI.

We thank for the suggestion and have added

"This functional has the virtue of providing very accurate cluster geometries at a relatively low cost (as discussed in the Supplementary Information),.."

6. Fig.2 is the basis of the structure assignment. However, it's not clear to me how exactly the EDOS shown is generated. In detail: pls. explain how the EDOS of the different isomers are scaled.

As usually done, we depict the EDOS by broadening the Kohn-Sham level distribution by Gaussians (of 0.03-0.04 eV width), in order to simulate the experimental broadening caused by spectrometer resolution as well as vibrational and lifetime broadening effects. We then align EDOS and PES so that the position of the lowest binding energy peak coincide (the necessary energetic shifts are given in Supplementary Table 4). The comparison of the EDOS and the PES is not absolutely straightforward, though, as the DFT calculation yields the energies of the Kohn-Sham states, but no detachment cross sections. Therefore, one can only compare peak positions, but not peak heights. The scaling of the EDOS of different isomers was done in a way to best visualize the agreement of the peak positions, but is not meant to reproduce the abundance of these isomers. In the SI we have now included fitted (but still only partially reliable) relative contributions of the isomers (see below).

7. Fig. 3: Alternative views of the clusters are provided for certain cluster sizes. Please indicate the relative orientation in the figure. When different isomers are shown, it is helpful to provide their relative energy.

We have now specified the relative orientations when two views of a single structure are provided. In these cases, we have added to the cluster size the letters f,s,t,b for front, side, top and bottom views, respectively. The caption has been revised accordingly (see below). Related to this issue, we have substituted one of the previous images of Au47- to show now a top view.
Finally, we would like to mention that all the relevant energy differences between isomers are already shown in Supplementary Table 6. As Fig. 3 is quite crowded with information and we have further increased the font sizes (which reduces the available space), we prefer to keep this numerical information in the SI. In the caption of Fig. 3 we now refer to the SI for the relevant relative energy differences.

8. Fig3: Some higher-lying isomers are necessary in order to explain the PES (Au25 II, Au32 II, III, and others). These are only shown in the SI. However, the reader should be able to follow the paper without constantly looking at the SI, so I suggest showing all the relevant structures in Fig.3

We agree that it is not optimal that the reader has to look up the structures of the higher isomers in the SI. Nevertheless, the main purpose of Fig. 3 is to explain the main structural motifs of gold cluster anions in the studied size range, with an emphasis on high symmetry geometries. For space reasons and in order not to make Fig. 3 look very crowded we think it can be justified to leave the additional structures in the SI. What we will do, though, is to provide the atom coordinates of all structures discussed, so that the interested reader can look at them in 3D.

9. P18 SI: The GM for Au39- seems significantly different from the structural motif of the PES fitting isomers II and III. This makes the "configurational entropy only" assumption questionable. Pls. comment.

It is true that the two structural families (of the GM on one hand and isomers II and III on the other hand) are very different, but they happen to be very close in energy. We have in fact done additional calculations (see below), which indicate that the C_{2v}-structure is indeed slightly less stable than the two other isomers. Nevertheless, the entropy argument remains valid; based on the energies all three isomers should be present in the experiment, but additionally taking the configurational entropy into account, the former "GM" should be quite strongly suppressed.

If we understand the referee correctly, he/she wants to point out that not only the configurational, but also the vibrational entropy should matter. This is fully correct; one can assume the two structural families to exhibit different details of the vibrational density of states, which could have a noticeable influence on their entropy. In order to estimate the resulting isomer population, one needs to know both the vibrational density of states and the "freeze in" temperature at which isomerization does not occur anymore on the timescale of the experiment. We have not performed the computationally rather intense calculation of the former yet, and we currently do not really know the latter. Therefore, indeed one has to be aware that inclusion of the vibrational entropy could change the prediction derived from the configurational entropy. In order to underline this, we have included the following sentence in the SI (end of Section 5):

"One should note that this discussion neglects possible differences in vibrational entropy, which could similarly influence the isomer population. If the vibrational density of states is significantly different for different isomers, the isomer with overall lower vibrational frequencies (leading to a larger heat capacity at low temperatures) will have a larger vibrational entropy around room temperature and therefore an enhanced equilibrium abundance. For isomers belonging to the same structural family one can assume rather similar vibrational frequencies; for very different families, however, this might not be the case. This is, therefore, an aspect which certainly warrants further work."

10. SI Table 4: When two isomers are required to fit the experimental data, what are their respective contributions?

We have followed this helpful comment and have now included the respective isomer contributions as obtained from the simple fitting in Supplementary Table 4. One should nevertheless emphasize that these are only rather approximate values, as the comparison between EDOS and PES does not take the state dependence of the photodetachment cross sections into account – peak positions can match very well, but peak heights generally do not.

Reviewer #4

1. For those clusters where the best agreement with the experimental PES arises from a low-energy isomer rather than the calculated ground state, such as Au27-, Au32-, and Au39-, have the authors verified that structural reordering does not occur when full relaxations are performed at the PBE0-SO-D3 level? None of these structures are included in Table S1, which shows the accuracy of the SP energy calculations on PBEsol-optimized geometries. Given the likely flatness of the potential energy surfaces in these cases, it would be important to confirm

that the use of different levels of theory for SP energies and geometry optimization does not lead to inconsistencies in energetic ranking or structural assignments.

We agree with the point of the referee. In cases of near-degeneracy, it might happen that a PBE0-D3 explicit optimization leads to some re-ordering of the energies of the involved isomers. What we claim, however (and we support this claim with the results in Supplementary Table 1 and the new Supplementary Table 2), is that the PBEsol optimal atomic coordinates are so close to the PBE0-D3 optimal atomic coordinates that near degenerate isomers at the PBE0-D3/PBEsol level will remain nearly degenerate at the PBE0-D3/PBE0-D3 level of theory. It is true that sizes 27, 32 and 39 were not included in Supplementary Table 1, and they should have been. We have performed explicit PBE0-D3 optimizations for these three sizes and also for N=35, which is another size for which two competitive structures exist.

The following table (Supplementary Table 2) summarizes the energy differences at PBE0-D3/PBEsol and PBE0-D3/PBE0-D3 levels of theory:

		PBE0-D3/PBEsol	PBE0-D3/PBE0-D3
N=27	GM (D6d)	0	0
	1st (Cs)	+0.070	+0.050
N=32	GM (D3h)	0	0
	1st (C2)	+0.001	+0.015
	2nd (C1)	+0.021	+0.032
N=35	GM (C3)	0	0
	1st (C1)	+0.028	+0.017
N=39	GM (C2v)	0	+0.007
	1st (C1)	+0.010	0
	2nd (C1)	+0.011	+0.003

For N=27, the difference between the GM and 1st excited isomer energies is reduced by 0.02 eV upon PBE0-D3 re-optimization. Therefore, nothing essential changes, but if the theoretically predicted equilibrium populations at room-T were 50%-50% based on the PBEsol geometries, now they are 36% for the D6d structure and 64% for the Cs structure. In summary, the new results reinforce the observation that it is the EDOS of the Cs isomer that should better match the PES, although with a non-negligible contribution from the GM geometry, which would be only partially "hidden" by the configurational entropy contribution.

For N=32, the symmetric (D3h) GM structure becomes even more stable upon PBE0-D3 reoptimization. Nevertheless, with the new energy differences we estimate equilibrium populations of 12,7% 42,8% and 44,5% for D3h, C2 and C1 isomers, respectively, i.e. the new results do not modify the observation that the D3h structure is a hidden GM structure and that the C2 and C1 structures dominate the photoemission spectrum of Au32-.

For N=35 the situation is similar to N=27: the more symmetric (C3) structure continues to be the (partially hidden) GM upon reoptimization. The new equilibrium populations are 39.2% and 60.8% respectively, so the less symmetric isomer is predicted to dominate the photoemission spectrum, albeit with a non-negligible contribution of the C3 GM structure.

For N=39, the twisted pyramids (1st and 2nd isomers) turn slightly more stable than the polyhedral C2v structure upon reoptimization, so here we have an example of what the referee suggests in his report: a slight re-ordering of isomers. Nevertheless, the essential message (that the 3 structures are nearly degenerate) is not modified. Neither the prediction that the two C1 isomers should dominate the photoemission spectrum, while the population of the C2v structure should be very low (around 10%), due to an unfavourable configurational entropy factor.

The new results are shown in Supplementary Table 2 in the new version of the SI and discussed in Subsection 1.3.

2. The authors suggest that their method is transferable to ligand-stabilized gold nanoclusters. Have the authors performed any validation with such systems? Could they comment on how ligands (for example, thiolates) might alter the structural trends they report? Even a single comparison with one of the most studied systems, such as Au25(SR)18- could support the claim of transferability.

In the paragraph mentioned by the referee, we just stated our expectations about the good performance of the PBE0-SO-D3 level of theory for more general systems containing gold, as for example gold clusters supported on a substrate, or ligand-protected (functionalized) gold clusters. We have tried to be completely clear on this aspect in the revised version of the paper. We believe that this is a sensible expectation, because (for example in a thiolate-protected gold cluster) gold is the most complex atomic species within the cluster, while the remaining atomic species (such as sulfur, carbon, nitrogen, hydrogen...) are known to be well described by hybrid functionals from many computational chemistry studies. So, the rationale for our expectation is: if we have identified a level of theory (PBE0-SO-D3) that accurately captures the physics of Au-Au interactions, it should continue to do so when the gold cluster is in contact with other atoms which are themselves well described by a hybrid functional. This is all we wanted to express in that paragraph, though we admit, as the referee points out, that we have not explicitly checked if our expectations hold. In our paper, we propose a three-level computational approach to efficiently describe pure gold clusters in the gas phase: (1) a fast scanning of the approximate potential energy surface generated by a Gupta or a machine-learning potential to identify candidate initial structures; (2) identification of a computationally unexpensive GGA functional (in our case, PBEsol) to be able to optimize the structures of hundreds of isomers for each separate size, and to obtain very accurate geometries; (3) single-point hybrid calculations at PBE0-D3 and PBE0-SO-D3 levels of theory, to obtain accurate properties for gold. In the paragraph mentioned by the referee, we were referring to point (3) only, although the two other points could of course be checked as well for supported or for ligand-protected gold clusters. For a thiolate-protected cluster, for example, step (1) could be done (with some dedicated work!) by parameterizing a machine-learning potential, or maybe a ReaxFF potential, as in recent work by the group of Hakkinen [Chem. Commun., 2020,56, 8087-8090]. Identifying a computationally inexpensive GGA that produces ``reasonable'' geometries (step 2) should be possible as well, but obtaining cluster geometries from a GGA calculation that are so accurate as to justify single-point calculations with the hybrid functional is not guaranteed, and should of course be checked for each separate system. For pure gold clusters, we have shown in this report that PBEsol does indeed produce sufficiently accurate geometries. This is a finding that is not expected a priori to be transferable to other systems: even if PBEsol describes the geometry of Au-Au bonds well, it will not necessarily produce accurate geometries for Au-S, S-C or C-H bonds, for example. Nevertheless, step (2) in our procedure is just a convenient one, but it is not a necessary step. If the GGA geometry is not good enough, one should just optimize the cluster geometry at the PBE0-D3 hybrid level (more expensive, but possible).

The referee also asks about how ligands (for example, thiolates) might alter the structural trends here observed. It is in fact expected that the thiolate monolayer will significantly alter the GM cluster

geometry. The Au25SR18 cluster mentioned by the referee is a clear example. The geometry of the Au-subsystem in Au25SR18 is completely different to that of Au25 in the gas-phase, because 12 of the 25 gold atoms in Au25SR18 are rather a part of the protecting shell than a part of the metallic core (these 12 atoms form 6 Au-Au dimers that surround the icosahedral core, and are involved in covalent bonds with sulfur atoms). The core itself is a 13-atom icosahedron, but according to all calculations it contains only eight delocalized electrons, so it should be seen as a Au_{13}^{5+} cation. The GM geometry of Au_{13}^{-} in the gas-phase is clearly not an icosahedron, because in gold anions an internal gold atom appears only at N=25. So, in summary, we expect that the GM geometry will strongly change when the cluster is protected by thiolates, due to the formation of new chemical bonds. Other, more general, trends identified here might persist within the core region of functionalized clusters (for example, the observation of frustrated icosahedral packing), but even those are not guaranteed. It would of course be interesting to see if some of the structural trends identified here are retained in monolayer-protected versions. As far as supported clusters are concerned, we would expect that if the gold clusters are softly landed on an inert substrate, they could retain their original structures to a large degree; but if reactive substrates are used, the structures are expected to be influenced by the new chemical bonds formed between Au and substrate atoms.

Following the recommendation of the referee, we have performed some calculations on the Au25SR18 cluster, with R=CH3 in order to make the spin-orbit calculations feasible within a reasonable time. This is a usual model ligand, which was considered in the recent work of Hakkinen's group as well [Chem. Commun., 2020,56, 8087-8090]. In that work, the authors report a first attempt at searching isomer configurations for this cluster in the gas phase, and they succeed in identifying several isomeric structures that are more stable (in the gas-phase) than the well-known crystal structure. As we are working with gas-phase clusters in our study, we have chosen the new topological isomer identified by Hakkinen's group in our test calculations. We notice that the new structure was later on observed experimentally [J. Am. Chem. Soc. 2021, 143, 1273−1277], though with a different, more complex, ligand. Doing extensive calculations on thiolate-protected clusters is obviously beyond the scope of this report, so we just present a small set of test calculations aimed at demonstrating that our computational settings can be applied to those clusters as well. Unfortunately, there are no photoemission data for the cluster that we calculate. We have just found one previous study, by Tsukuda and coworkers [Nanoscale, 2017, 9, 13409–13412] showing a photoemission spectrum of Au25SR18 in the gas phase, but the authors employed R=C_12H_25 as a ligand, so that the cluster contains more than 700 atoms and the calculations including spin-orbit effects would be extremely time consuming. A previous work by Jung et al. [Nanoscale, 2012,4, 4206-4210] has shown that the vertical detachment energies can be very sensitive to the specific ligand, so our results are not directly comparable to experiment. Clearly, more photoemission experiments are needed to make an EDOS/PES comparison viable for these systems. We have taken the geometry of the new topological isomer of Au_25(SCH_3)18 directly from Hakkinen's work. In that paper, a PBE optimization is performed. We have done PBE, PBEsol and PBE0-D3 geometry optimizations of that initial structure, and then performed PBE0-D3 single-point calculations on both the PBE and PBEsol local minima. The results are summarized in the following table. Two types of gold atoms are distinguished: AuC refers to atoms within the icosahedral core, while AuL refers to the 12 gold atoms in the ligand shell.

Within the Au13 inner icosahedron, we additionally distinguish between radial (first number) and tangential (2nd number) bond lengths. All the distances are to be understood as average distances over the corresponding set of bonds. Delta(E) is the energy difference with respect to the PBE0-D3/PBE0-D3 calculation, in eV units.

Level of theory	Delta(E)	d(AuC-AuC)	d(AuC-AuL)	d(AuL-AuL)	d(AuC-S)	d(AuL-S)	d(S-C)	d(C-H)
PBE0-D3/PBE	2.25	2.832/2.974	3.645	3.102	2.395	2.346	1.848	1.095
PBE0-D3/PBEsol	0.81	2.781/2.923	3.326	3.176	2.368	2.322	1.832	1.105
PBE0-D3/PBE0-D3	0	2.777/2.918	3.341	3.182	2.345	2.307	1.810	1.091

One first observation is that the PBE0-D3 optimal geometry differs quite a lot from the optimal PBE geometry, but is much closer to the PBEsol optimal geometry. This is also seen in the energies obtained in the single-point calculations: the energy of the PBEsol geometry is much closer to the fully consistent PBE0-D3 energy, so the PBEsol geometry is a much better starting point for PBE0-D3 optimizations than the PBE one. Our PBE0-D3 optimization was in fact started from the PBEsol geometry, and converged in about 50 ionic steps only.

Similarly to the pure gold clusters, the PBEsol geometry continues to be very close to the optimal PBE0-D3 geometry. Therefore, in any PBE0-D3 treatment of thiolate-protected clusters, we can recommend that a pre-optimization be performed with the much cheaper PBEsol functional. The PBEsol geometry, as expected, is not as good as for pure gold clusters, i.e. not as good as to warrant a single-point PBE0-D3 calculation if a very accurate geometry is needed. We notice that all Au-Au bond lengths preserve the impressive accuracy seen in pure gold clusters, so at least the geometry of the metal core can be trusted at the PBEsol level. PBEsol also improves Au-S and S-C distances, and only worsens C-H distances. The re-optimization at PBE0-D3 level essentially fixes the geometry of the ligand shell without significantly affecting the geometry of the metallic core.

We finally notice that the PBE geometry predicts too long distances between gold atoms in the core and gold atoms in the ligand shell, and expanded Au-Au lengths in general. This is a well-known deficiency of PBE for gold clusters.

Finally, we have performed single-point PBE0-SO-D3 calculations on both the PBEsol and PBE0-D3 optimal geometries. The calculations, shown in figure below, demonstrate, on the one hand, that S-O effects continue to have essential effects on the EDOS, so we confirm that spin-orbit effects should be employed if an accurate representation of the electron density of states of thiolate-protected clusters is needed. On the other hand, the figure also shows that the EDOS generated from PBE0-SO-D3 calculations at the PBEsol geometry is already very close to the more correct one obtained at the PBE0-D3 geometry. Even if the situation is not as perfect as for the pure gold clusters, the PBEsol geometry can be sufficient for a meaningful assessment of the structures of functionalized gold clusters via an EDOS/PES comparison, as the main differences occur only at higher binding energies.

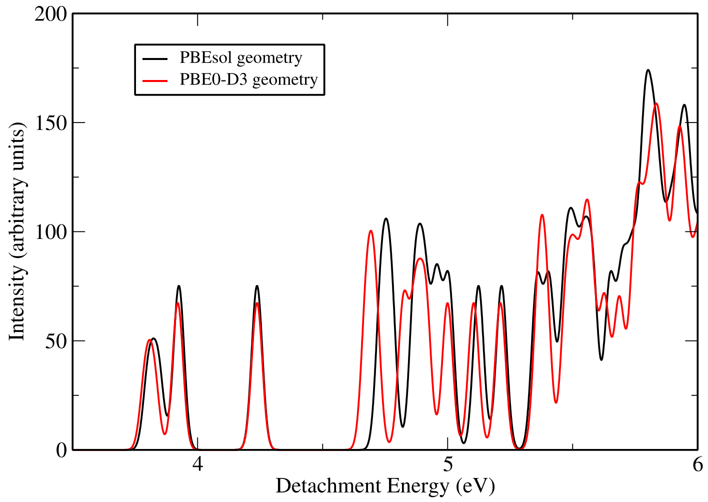

These are in fact interesting findings, and we will further exploit them in future work. Therefore, we would prefer not to include these calculations in the SI, but to show them in a later publication.

3. Regarding the figures:
   a) Figures showing the comparison of EDOS vs PES could be improved by adding a quantitative error measure. Adding vertical lines for key peak positions would also aid the reader.

We would like to point out that in Section 4 of the Supplementary Material we already have presented a quantitative assessment of the EDOS/PES agreement, with results given in Supplementary Figure 7 and Supplementary Table 6. As can be seen in Supplementary Figure 7, the fits obtained are not perfect, as theoretical peak heights differ from the experimental ones (the EDOS does not take detachment cross sections into account). One could improve the fit by allowing different heights of the different Kohn-Sham levels, but this makes the fitting very unstable in cases where the EDOS/PES agreement is not good.

We did not try this as already with this crude fitting approach in most cases the EDOS of the lowest energy structure presented in Fig. 2 clearly yields the smallest fitting error χ^2. In cases of "hidden" ground states the EDOS of the next higher (or of combinations of first and second or second and third isomer) yield better fits. Size 60 is special, as only isomer V can reproduce the third (small) peak in the spectrum – here a combination of I and V has been used for the fitting. As mentioned above, we have now added the fitted contributions of the isomers to Supplementary Table 4. We would like to stress that this is the first time such a quantitative assessment of the EDOS/PES agreement has been performed in cluster physics.

With respect to the interesting suggestion of adding lines to the graphs, we have tested this, but felt that it leads to an even more congested look of the figure, so we decided not to follow the referee's advice in this case.

b) Labels for symmetry groups in Fig.3 are hard to read, and the color scheme for atom distinction is inconsistently described. The figure caption should be improved, and clearer labeling (with a higher font size) for the structures would be better.

We thank for this remark – indeed for size 41 the color red was used for a surface atom (and not for core atoms as described in the caption). Additionally for this size the core atoms were not colored. We corrected the figure.

Furthermore, we have increased the font size, and have modified the caption to better explain the color coding:

"The cluster size and the approximate point group symmetry of the structure are given below each image; the letters f,s,t,b indicate front, side, top and bottom views in cases more than one view is shown. The GM structures that are "hidden" in the experiment due to an entropic advantage of isomers with lower symmetry are marked with the letter h (for size N = 39 isomer III is practically degenerate with isomers I and II, but "hidden" due to its higher symmetry). The structure of size N = 41 is not the GM, but represents a structural motif seen for smaller sizes, e.g. N = 35. Red color is used for core atoms, while blue and green colors highlight some structural features at the cluster surface which are discussed in the text. Additional isomers are presented in the Supplementary Information, as well as the calculated relative energies of the isomers."

4. Consider uploading xyz coordinates as part of the SI material.

We have added xyz files for all structures shown in Fig. 3 and Supplementary Fig. 5 to the SI material.

We thank the referees again for their detailed and helpful comments. In the following we describe the changes we made to the manuscript and the SI.

Reviewer #1

There are no further issues with the response to the review comments or the revised manuscript. The necessary revisions have been adequately addressed, and I am satisfied with the changes. Therefore, I agree to accept it for publication.

Reviewer #2

I appreciate the revision work done by the authors. The bibliography in the main text should include the references suggested.

Probably the manuscript will befit better the Nature style if the authors add a pictorial representation of the numerical workflow.

Minor English typos and syntax that can be done during the proof reading

We thank for these comments. We have now included the two references given in our reply letter in the main text.

We thought about how to depict the numerical workflow, but did not find a convincing solution, so we refrained from adding a figure.

With respect to typos and syntax, we hopefully have corrected most of them.

Reviewer #3

In the revised version the authors have answered my questions and addressed my concerns, and I believe the same applies to the other reviews. I support publication in Nature Communications; the article can be published basically in its current form. Only a few minor points remain:

1. Pls. update the data availability statement with regard to the coordinate files.
2. Page 22 SI: 'isomers II and III ... present in approximately equal amounts...' is inconsistent with SI. Tab. 6 : 'Au32-: II+III (0.1, 0.9)'
3. SI: pls. check citation of reference [13]

1. We have modified the data availability statement to:

"Source data and coordinates of all cluster structures presented are provided with this paper in the Supplementary Information data files SourceData.tar and Supplementary Data Structures.tar."

2. This is correct. We have changed the paragraph to:

"Thus, one expects that the population of the highly symmetric GM is reduced by entropy: isomers II and III should be the dominant species in the experimental sample, present in approximately equal amounts, while both IV and the true GM should contribute comparatively less to the PES. Indeed Fig. 2 suggests that isomer I is not dominant, the spectrum can be fully explained by contributions from isomers II and III. Isomer III turns out to be more abundant than isomer II, which hints at a slightly higher energy of isomer II than the one calculated or at an additional influence of vibrational entropy."

3. That was helpful, we have now corrected the reference (it was arXiv:2003.08879 (2020))

Reviewer #4

The authors have adequately addressed most of my concerns by including new data with a very detailed discussion. Also, new adjustments in the text and figures clarify their interpretation of the results. Thus, I recommend the manuscript for publication in its current form.
